# Metal–Phenolic Complexation Governs Soil Mineral Crystallinity to Enhance Crop Growth

**DOI:** 10.1002/advs.77994

**Published:** 2026-09-30

**Authors:** Jong‐Rok Jeon, Omid Mazaheri, Tianzheng Wang, Ali Zavabeti, Ho Young Yoon, Nguyen Thanh Phong, Eun‐Nam Joe, Zhixing Lin, Shuaijun Pan, Chan‐Jin Kim, Frank Caruso

**Affiliations:** ^1^ Department of Chemical Engineering The University of Melbourne Parkville Victoria Australia; ^2^ ARC Research Hub for Smart Fertilisers The University of Melbourne Parkville Victoria Australia; ^3^ Division of Applied Life Science (BK21Plus) Gyeongsang National University Jinju Republic of Korea; ^4^ Department of Applied Life Chemistry Gyeongsang National University Jinju Republic of Korea; ^5^ Institute of Agriculture and Life Science Gyeongsang National University Jinju Republic of Korea; ^6^ Department of Chemical and Environmental Engineering School of Engineering RMIT University Melbourne Victoria Australia; ^7^ Department of Chemical Engineering Gyeongsang National University Jinju Republic of Korea

**Keywords:** crop nutrition, metal–phenolic complexes, plant polyphenols, plant secondary metabolites, soil inorganic components

## Abstract

Plants release polyphenols into soil under stress, yet their role in regulating soil mineral structure and nutrient availability is unclear. Herein, tannic acid (TA), a representative plant‐derived polyphenol, is used to first demonstrate the role of natural mineral particle restructuring in soil to control nutrient availability—a phenomenon that is facilitated through the formation of amorphous metal–phenolic complexes. From these findings, a coordination‐driven assembly strategy is devised to engineer minerals with tunable crystallinity via coprecipitation of TA and minerals (calcium phosphate and struvite). These nature‐inspired hybrid minerals exhibit reduced crystallinity (up to 46% relative to the control minerals) depending on the amount of TA incorporated (up to 17%), enabling tunable disassembly behavior. The TA‐loaded minerals exhibit enhanced nutrient release and polyphenol codelivery under biologically relevant conditions, primarily driven by TA‐induced destabilization of the mineral lattice. Pot experiments reveal increased biomass production (up to 2.5‐fold) using the TA‐loaded minerals, largely due to enhanced nutrient bioavailability, stimulatory effects of TA on crop growth, and enrichment of crop‐beneficial microbes. The findings unveil the role of plant polyphenols in regulating soil mineral dynamics and demonstrate a strategy for designing fertilizers that leverage plant–soil–microbe feedback mechanisms.

## Graphical abstract

A coordination‐driven, coprecipitation approach is presented to obtain polyphenolic‐incorporated minerals with tunable crystallinity, offering insights into how plants may influence soil mineral structures. Soil minerals can incorporate polyphenolic secondary metabolites and enhance plant nutrition by facilitating the formation of amorphous metal–phenolic complexes.

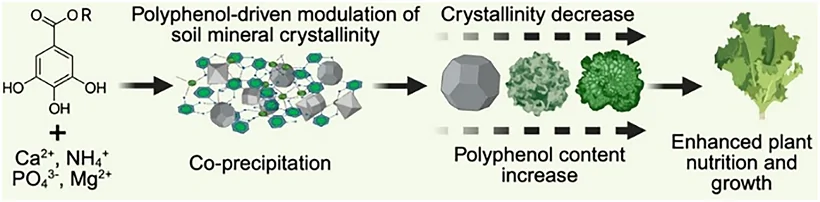

## 1 Introduction

A substantial amount of photosynthetically fixed carbon (up to 20%) in plants is known to be secreted into soils through the root systems. Root exudates contain primary (e.g., sugars and organic acids) and secondary (e.g., polyphenolics) metabolites depending on plant nutrition and stress states [1, 2, 3, 4, 5]. Sugars and organic acids serve as labile carbon sources for soil heterotrophic microbes, leading to the selective enrichment of the soil microbiome [5]. In addition, the strong metal‐chelating capacity of organic acids destabilizes soil mineral structures and promotes their dissolution [6].

Plant polyphenolic secondary metabolites are produced and secreted when plants are exposed to stress conditions [7, 8, 9]. Unlike primary metabolites, polyphenolic secondary metabolites function by inducing the expression of stress tolerance genes and inhibiting pathogen infections, thereby helping plants to respond to both biotic and abiotic stresses [10, 11]. For example, the jasmonic acid–isoleucine signaling pathway, which plays a key role in gene expression related to drought and pathogen defense, is activated in the presence of polyphenols [12]. The inhibition of pathogen proliferation by plant‐derived polyphenols has been experimentally demonstrated [13]. Moreover, the secreted polyphenols can facilitate the growth of specific soil microbes that can be beneficial for plant growth and stress defense [7, 14, 15, 16].

Soil contains various metal‐containing minerals, including calcium phosphate (CaP), ferric oxide, iron phosphate, and aluminum hydroxide, which are essential for soil functions such as plant nutrition. Plant‐secreted polyphenolic compounds likely interact with these minerals through metal–catechol coordination [17, 18, 19], given the adhesiveness of polyphenols on diverse mineral particle surfaces [20, 21, 22, 23]. Such adhesive interactions may further facilitate metal leaching, as demonstrated by the use of TA for metal recovery [24, 25]. Moreover, metal ions dissolved from soil minerals in the rhizosphere may also interact with polyphenols, forming metal–phenolic complexes, thus affecting the crystallinity of newly formed minerals, potentially facilitating micronutrient mobilization and plant uptake and also changing nutrient mobilization and plant availability [26, 27, 28]. Mineral association could protect organic compounds from microbial degradation by reducing their accessibility to extracellular enzymes [29, 30]. Polyphenols could also inhibit soil enzymes, and such interactions may contribute to the persistence of plant‐derived polyphenols in soil [31]. We therefore hypothesize that mineral‐associated polyphenols could potentially provide a chemical record of previous plant stress and affect plant–soil interactions. To understand this, the effects of polyphenolic metabolites and metal–phenolic coordination on the structure (e.g., crystallinity) and function of naturally occurring soil minerals and the implications of this process for nutrient cycling and plant nutrition need to be understood [32, 33]. Furthermore, developing strategies that can exploit the beneficial effects of polyphenolic metabolites to modulate mineral crystallinity and functions could offer a pathway to improving the performance of mineral fertilizers.

Herein, we first investigated the influence of plant polyphenolic metabolites on the restructuring of soil minerals through the formation of metal–phenolic complexes in soil and the effects of this complexation on associated plant nutrition (Scheme 1). Tannic acid (TA) was selected as the model plant polyphenolic secondary metabolite. Soil samples were treated with TA to confirm the formation of metal–phenolic complexes in situ. The metal and phosphate (P) contents as well as the fluorescence emission–excitation matrices (FEEMs) of their leachates were compared with those of untreated soil samples. Thereafter, the P and phenolic contents of the alkali‐precipitated mineral‐like particles derived from the leachates were assessed to determine the incorporation of metal–phenolic complexes. Secondly, inspired by the findings of the in situ metal–phenolic complexation studies, we engineered two types of hybrid mineral particles via a coordination‐driven assembly strategy involving TA and mineral particles (i.e., CaP and struvite). CaP and struvite were selected as model minerals because their P‐rich (CaP) and P,N‐rich (struvite) compositions are directly relevant to plant nutrition and fertilizer development. Moreover, both CaP and struvite are recognized as alternatives to conventional chemical fertilizers [34, 35]. Following the incorporation of TA during the wet precipitation of CaP and struvite, structural and compositional analyses were conducted to visualize the formation of metal–phenolic complexes within the mineral particles and to assess associated changes in the crystallinity of the mineral particles. The dissolution behaviors of the TA‐loaded fertilizer particles in distilled water (pH 6), soil organic matter (i.e., leonardite‐derived humic acid)‐containing, citric acid (pH 4)‐acidified, and acetate buffered (pH 5.1) water were evaluated to establish the relationship between metal–phenolic complexation and the release of plant nutrients and TA. Finally, lettuce was cultivated in soil pots treated with TA‐loaded fertilizer particles, and the growth rate and soil microbiome were analyzed. The broader applicability of our approach was demonstrated by the cultivation of two additional crops, pak choi and corn. Our insights into the roles of plant secondary metabolites offer opportunities for their use in agriculture. Furthermore, given that TA is derived from renewable plant sources and commonly found in soils, our method of incorporating TA presents a practical strategy for developing soil‐compatible and nontoxic mineral fertilizer alternatives.

**SCHEME 1:**
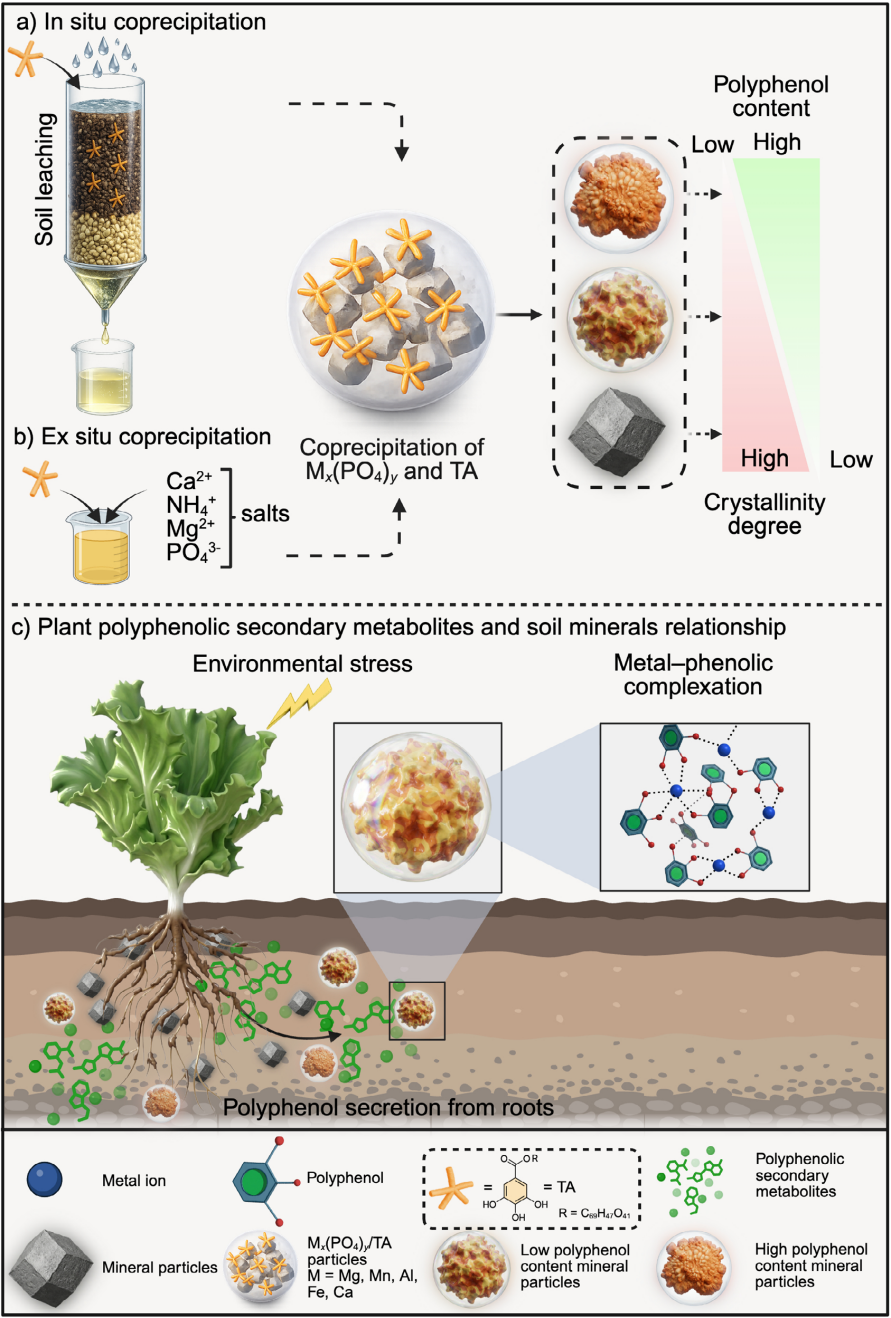
Schematic of the formation of TA‐mineral particles via coprecipitation of TA and metal ions to form metal–phenolic complexes. (a) In situ coprecipitation of polyphenol‐loaded minerals in soil. (b) Ex situ coprecipitation of polyphenol‐loaded minerals to develop fertilizers. (c) Proposed mechanism of the relationship between secreted plant polyphenolic secondary metabolites and soil minerals. The scheme was created in BioRender.com. Caruso, F. (2026) https://BioRender.com/rk2erge. The chemical structure of TA is provided in Figure S1.

## 2 Results and Discussion

TA was first introduced into soil (collected from Jinju area in South Korea) using the in situ coprecipitation method via leaching studies to understand the mobilization of mineral components through dissolution (Scheme 1a). Soil leachates (using distilled water) were collected to analyze and quantify the metal ions and P by inductively coupled plasma‐optical emission spectroscopy (ICP‐OES). In the leachate of the soil control sample (TA‐free soil), Al and Mn were below the detection limit but were detected at concentrations of approximately 10 ppm in the leachate of the TA‐containing soil (Figure 1a). Other metals, including Ca, Fe, and Mg, were also detected at concentrations up to 15 times higher in the leachate of the TA‐containing soil than in the control. Furthermore, P was present at levels up to 22 times higher in the leachate of the TA‐containing soil than in the control (Figure 1a). These findings suggest that both metals and P retained in soil minerals can be effectively dissolved into the soil leachates by TA. To eliminate the influence of organic matter and evaluate the effect of only the incorporated TA, soil samples treated at 600°C were examined. The same trend was observed in the soils treated at 600°C (Figure 1d), although the leaching enhancement was lower than that observed in non‐heat‐treated soils (Figure 1a). The catechol moieties in TA are known to chelate metals [21, 22, 23], which may contribute to the destabilization of soil mineral structures and thereby accelerate mineral dissolution. Heating at 600°C reshapes soil mineral states and may strengthen interactions between P and metals within the minerals, which could account for the reduced effectiveness of TA‐driven metal ion/P leaching [36, 37].

**FIGURE 1:**
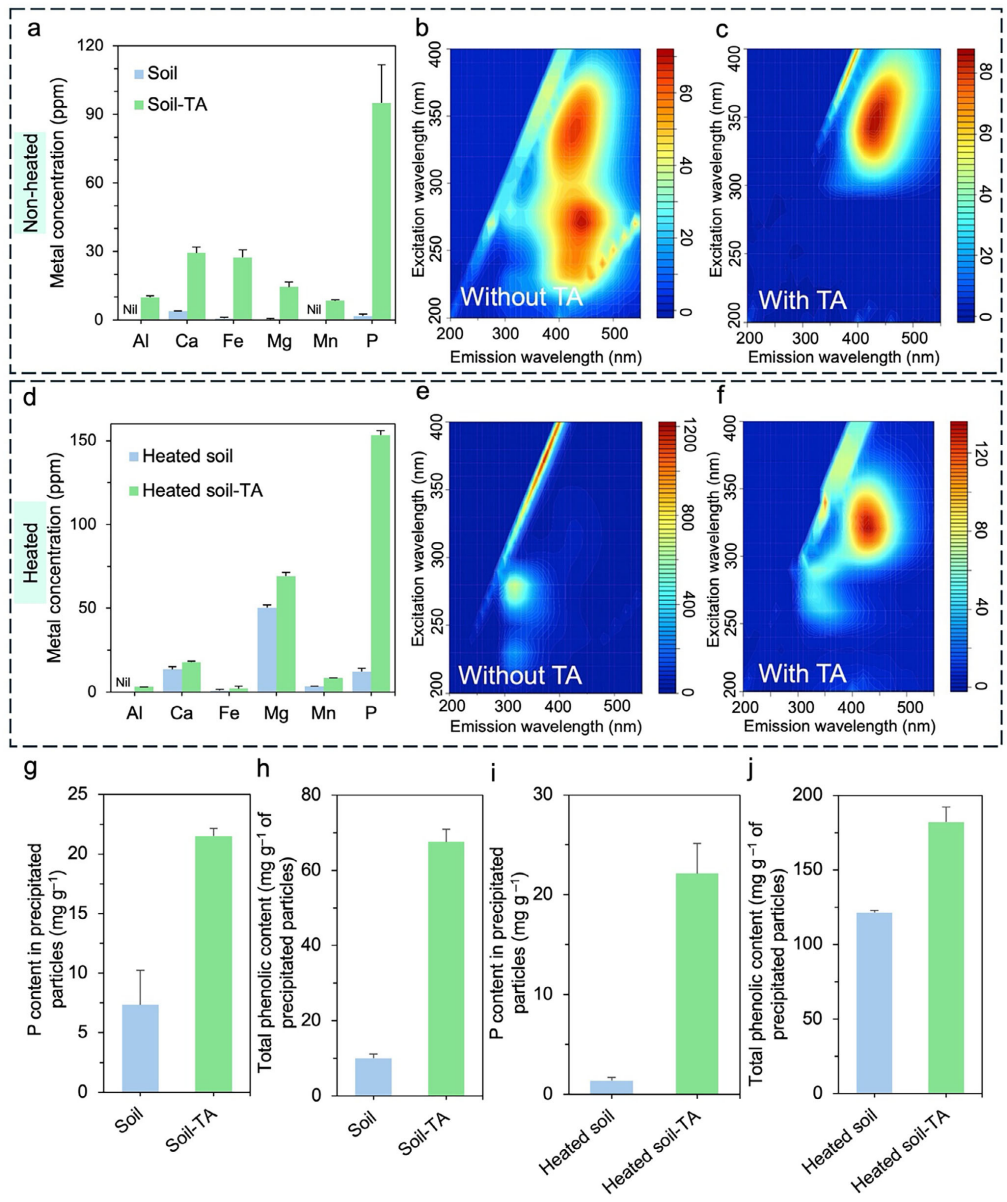
Role of plant secondary metabolites on the availability of metals and P in soil. (a,d) Metal and P contents in non‐heat‐treated (a) and heat (600°C)‐treated (d) soils with and without TA. (b,c,e,f) FEEM images of leachates of soil subjected to no heat treatment (b,c) and to heat treatment at 600°C (e,f) with and without TA. (g–j) P and phenolic contents of mineral‐like particles derived from leachates of non‐heat‐treated (g,h) and heat (600°C)‐treated (i,j) soils with and without TA. In (a,d) and (g–j), data are presented as means ± standard deviations (*n* = 3).

FEEM imaging was performed on the soil leachates. The soil leachate obtained from the TA‐free soil displayed a broad fluorescence region with excitation wavelengths of 225–375 nm and emission wavelengths of 350–475 nm (Figure 1b). In contrast, the soil leachate obtained from the TA‐containing soil exhibited a narrower and redshifted fluorescence region with excitation wavelengths of 310–400 nm and emission wavelengths of 375–525 nm (Figure 1c). The soil leachates obtained from the heat‐treated soils displayed a more pronounced redshift (Figure 1e,f). In the absence of TA, a weak fluorescence region with excitation wavelengths of 260–280 nm and emission wavelengths of 300–350 nm was detected (Figure 1e), largely due to the elimination of soil organic matter. In the presence of TA, a distinct and redshifted fluorescence region with excitation wavelengths of 300–350 nm and emission wavelengths of 400–474 nm was observed (Figure 1f). Compared with the FEEM image of TA dissolved in distilled water, that of the soil leachates obtained from TA‐treated soils was also redshifted (Figure S2). As metal–organic complexation is known to induce fluorescence redshifts, coordination between leached metals and TA may influence the results obtained from the FEEM images [38].

The contents of phenolic compounds and P in the mineral‐like particles derived from the soil leachates were assessed to examine whether the introduced TA was incorporated into the mineral structures (Figure 1g–j). For the non‐heat‐ and heat‐treated soils, particles derived from the TA‐driven leachates exhibited up to 6‐fold and 1.5‐fold higher phenolic signals, respectively, than those obtained for particles derived from TA‐free leachates. P accumulated at levels 4–10 times higher in particles formed from the non‐heat‐ and heat‐treated soil leachates in the presence of TA compared with those formed in the absence of TA. This behavior may be attributed to the preferential precipitation of metal phosphates over metal hydroxides, as TA enhances the leaching of P from soils. The amount of TA used in this soil study (Figure 1) is higher than that of typical soil conditions and has been selected primarily as a proof‐of‐concept to demonstrate TA‐mediated mineral mobilization and to generate sufficient mineral precipitates from the soil leachates for subsequent analyses. Therefore, the extent of mineral dissolution observed should not be directly extrapolated to environmentally relevant TA concentrations. Nevertheless, the results demonstrate the potential for TA‐mediated incorporation into P‐containing minerals within localized soil microenvironments surrounding plant roots.

Overall, the results in Figure 1 indicate that polyphenolic metabolites released from plant roots under abiotic and biotic stress conditions facilitate the dissolution of metal and P in soils and have the potential to reshape soil mineral structures by incorporating phenolic metabolites and recruiting additional P. Given that organic matter complexed with minerals is generally stable in soils—hydrophilic moieties that are susceptible to microbial decomposition are shielded by inorganic mineral components [39]—the observed phenomena suggest that plant stress conditions can be preserved in polyphenol‐loaded soil minerals as a form of soil memory (Scheme 1c). Most studies have primarily examined secondary metabolite‐driven modulation of microbial dynamics in the rhizosphere [4, 15, 40]. In this context, establishing a connection between secondary metabolites and their role in soil mineral reshaping will offer insights into long‐term plant stress defense mechanisms in soils. It could be valuable to explore how this mechanism confers advantages to subsequent plant generations.

To assess whether polyphenol‐loaded soil minerals are beneficial for plant nutrition, TA was incorporated into CaP particles via an ex situ coprecipitation method (i.e., the alkali wet‐precipitation method) to produce CaP–TA particles (Scheme 1b). Because CaP can be synthesized through alkaline pH‐induced precipitation of their constituent ions, pH 10.4 (unless otherwise specified, i.e., Figure S3) was selected as a representative alkaline condition to assess the effect of TA incorporation under different precipitation conditions. Compared with the white CaP particles, the TA‐loaded CaP particles were brown, with the color intensity increasing as the amount of TA increased. The weight losses (4%–23%) observed between 200°C and 600°C from thermogravimetric analysis (TGA) of the CaP–TA particles were directly proportional to the amount of TA (i.e., 0.5%–15%) added during the precipitation process, indicating that TA incorporation into CaP particles depends on the TA concentration in aqueous solutions (Figure 2a,c and Figure S4a). Derivative thermogravimetric (DTG) analysis revealed a thermal degradation peak of TA at approximately 270°C (Figure S4b). The peak slightly shifted to higher temperatures with increasing TA amounts used during precipitation, indicating enhanced thermal stability (Figure 2b). This shift was likely derived from quantitative differences in coordination between deprotonated catechol groups of TA and calcium ions under alkaline conditions during precipitation.

**FIGURE 2:**
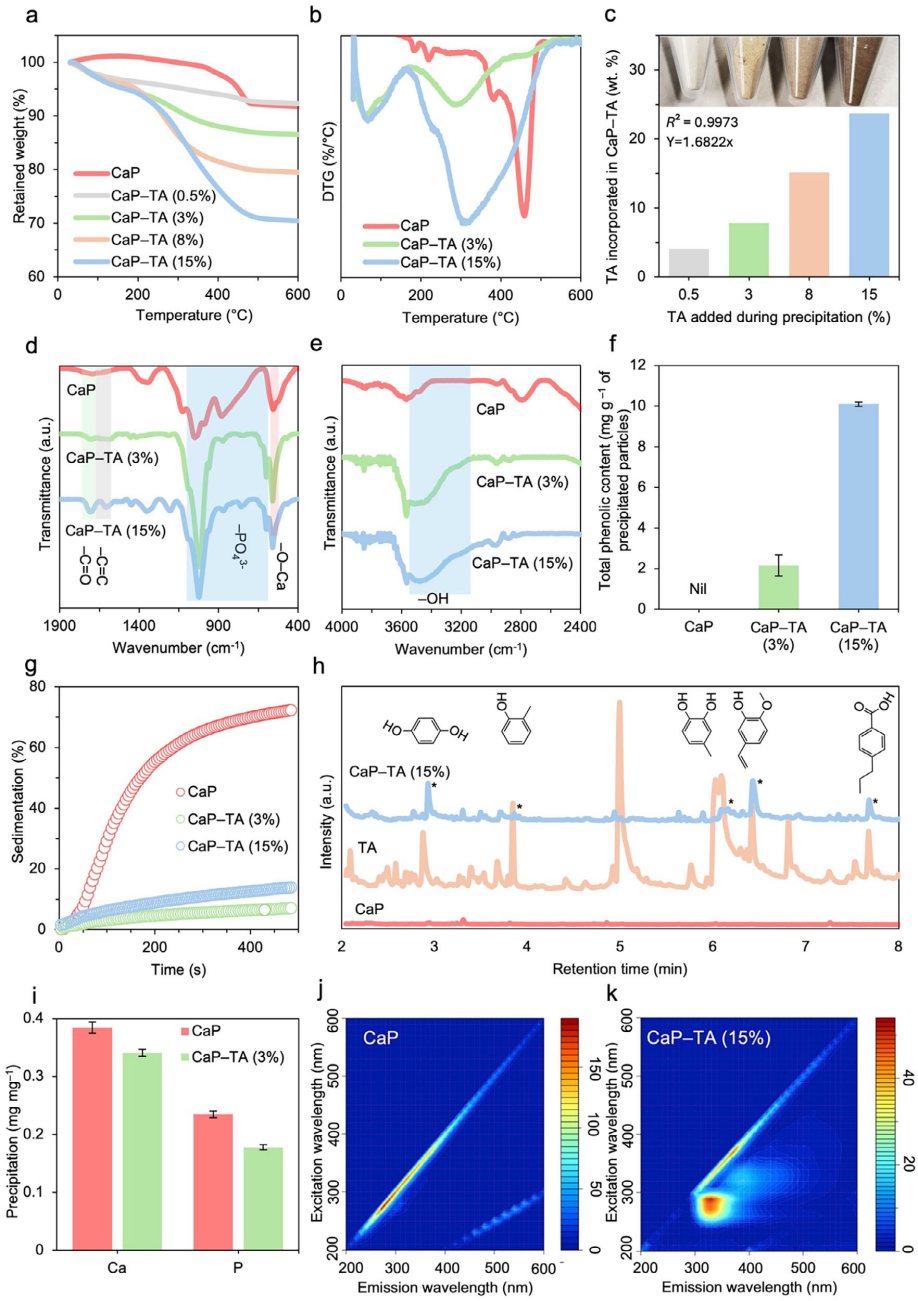
Loading of TA in CaP minerals. (a,b) TGA (a) and DTG (b) analysis curves of CaP and CaP–TA particles with different amounts of incorporated TA. (c) Amount of TA loaded in CaP–TA particles determined from TGA measurements from 200°C–600°C as a function of TA amount added during precipitation. (d,e) FTIR spectra of CaP and CaP–TA particles shown in the range of 1900–400 cm^−1^ (d) and 4000–2400 cm^−1^ (e). (f) Total phenolic content in CaP and CaP–TA particles. (g) Sedimentation of CaP and CaP–TA particles in water. (h) Pyrolysis–GC/MS chromatograms of TA, CaP, and CaP–TA (15%). (i) Ca and P elemental contents in CaP and CaP–TA (3%) particles quantified by ICP‐OES. (j,k) FEEM spectra of CaP (j) and CaP–TA (15%) (k). In (f) and (i), data are presented as means ± standard deviations (*n* = 2).

The Fourier transform infrared (FTIR) spectra of both the CaP and CaP–TA particles showed strong transmittance peaks at 887 and 1022 cm^−1^, corresponding to phosphate groups (Figure 2d) [41]. The FTIR transmittance peaks at 3451, 1712, 1606, and 500–620 cm^−1^ were attributed to hydroxyl, carboxylic groups, benzene rings, and Ca‐coordinated oxygens of TA, respectively (Figure 2d,e) [42, 43]. These peaks were only observed in the CaP–TA particles, confirming the incorporation of TA in the precipitated CaP–TA particles. The total phenolic content in the CaP–TA particles was dependent on the TA amount added during precipitation (Figure 2f). Furthermore, water sedimentation kinetics were significantly reduced with TA incorporation (Figure 2g), likely due to increased hydrophilicity of the CaP particles and the formation of a thicker solvation layer. Pyrolysis–gas chromatography–mass spectrometry (GC/MS) was applied to identify TA‐derived organic moieties involved in TA incorporation in CaP (Figure 2h). Mono‐phenolic and catechol‐type moieties originating from TA were detected in the chromatogram of CaP–TA, suggesting that the complex coordination plays a key role in TA incorporation. In addition, a carboxylic acid moiety (see the peak corresponding to 4‐propylbenzoic acid) was identified, supporting the partial involvement of carboxylate–metal coordination. Elemental analysis indicated that the CaP particles had a calcium‐to‐phosphorus (Ca:P) mass ratio of approximately 1.6:1. Upon incorporation of 3% TA, this ratio increased to 1.9:1 (Figure 2i). Specifically, the calcium content decreased by ∼10%, while the phosphorus content decreased more substantially by ∼25%. This change in the Ca:P ratio can be attributed to the integration of TA into the particle structure, which likely interferes with the stoichiometry of the CaP lattice. The phenolic groups in TA may preferentially bind to calcium ions [44], thereby recruiting additional metallic ions into the native lattice. This interaction suggests that TA is not only embedded within the mineral matrix but also plays a role in modulating the mineral phase composition. Differences between the FEEM images of CaP and CaP–TA dissolved in 0.1 N HCl revealed the formation of fluorophore species associated with CaP–TA components (Figure 2j,k). Compared with the FEEM image of TA alone in distilled water (Figure S2), that of CaP–TA displayed a relative enhancement in the fluorescence region with excitation wavelengths of 250–280 nm and emission wavelengths of 300–350 nm, which is attributed to Ca–TA coordination.

X‐ray photoelectron spectroscopy (XPS) data in Figure 3a revealed the presence of Ca─O coordination bonding in the CaP–TA particles, as indicated by a shift in the calcium‐related binding energy peaks toward higher values upon coprecipitation with TA. These shifts indicate a redistribution of electron density, where the calcium ions undergo partial electron withdrawal due to coordination with the electron‐rich oxygen atoms present in the phenolic groups of TA [45]. The C 1s XPS spectrum of the CaP–TA particles exhibited multiple characteristic peaks of TA (Figure 3b), with peaks at 284.6 eV (aromatic C─C/C─H bonds), 286.2 eV (C─O from phenolic and ether groups), and 288.6 eV (ester or carbonyl C═O bonds). A slight shift in the C═O peak was observed (compared to the C 1s XPS spectrum of TA [45]) upon coordination of the catechol groups and Ca^2+^ ions [46].

**FIGURE 3:**
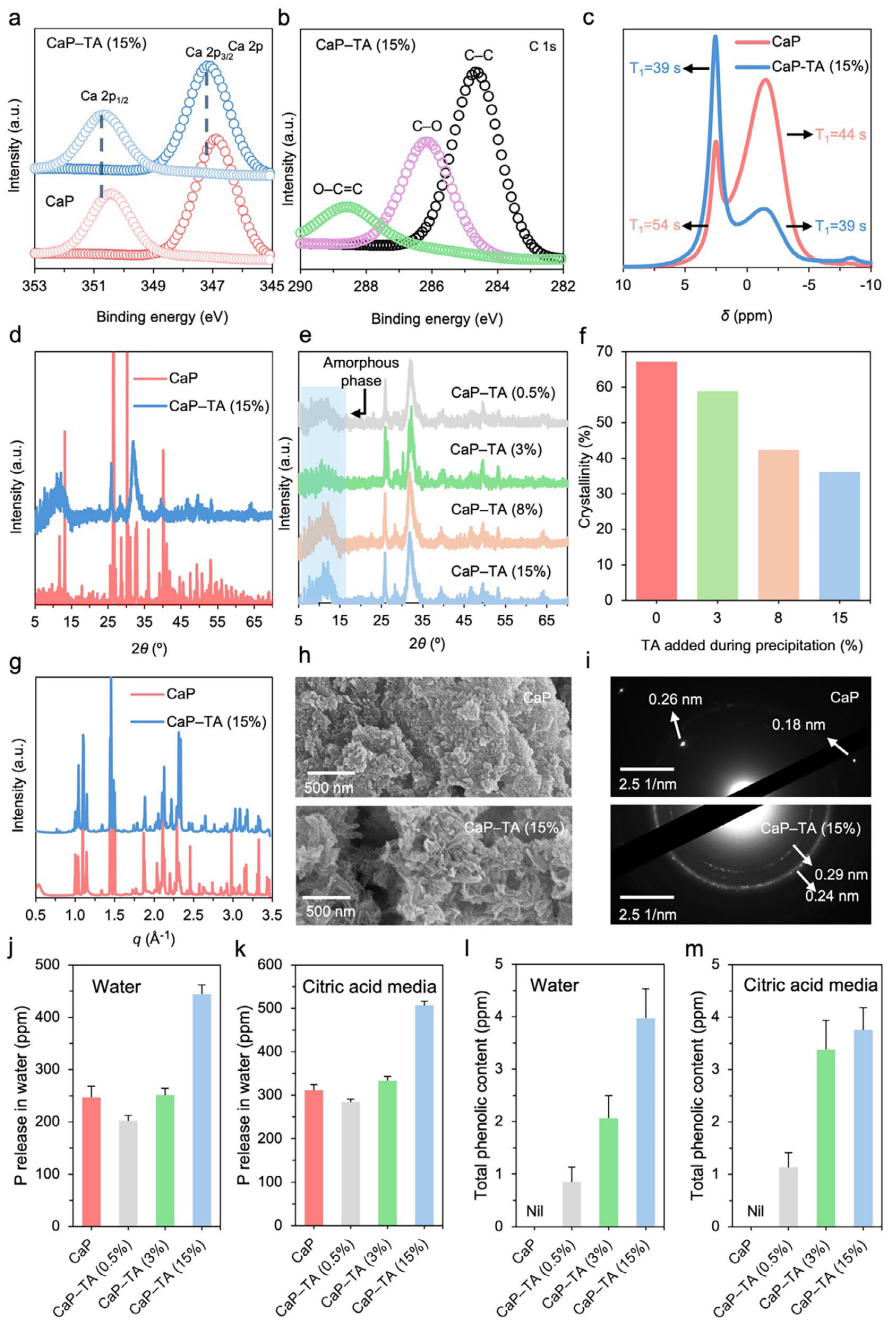
Characterization of CaP and CaP–TA particles. (a,b) XPS spectra of Ca 2p (a) and C 1s (b) of CaP and CaP–TA (15%) particles. (c) ^31^P solid‐state MAS NMR spectra of CaP and CaP–TA (15%); T_1_ is ^31^P saturation‐recovery. (d) XRD spectra of CaP and CaP–TA (15%) particles. (e) XRD spectra of CaP–TA particles with varying amounts of TA. (f) Crystallinity of CaP and CaP–TA particles measured from the XRD spectra in (d) and (e). (g) GIWAXS spectra of CaP and CaP–TA (15%) particles. (h,i) SEM images (h) and SAED patterns (i) of CaP and CaP–TA (15%) particles. (j–m) Release of P (j,k) and phenolic compounds (l,m) from CaP or CaP–TA particles in distilled water (pH 6) (j,l) and citric acid‐acidified water (pH 4) (k,m). In (j–m), data are presented as means ± standard deviations (*n* = 3).

To probe the local phosphate environments, ^31^P solid‐state magic‐angle spinning nuclear magnetic resonance (^31^P solid‐state MAS NMR) and T_1_ relaxation measurements were performed (Figure 3c and Figure S5). TA incorporation altered the ^31^P spectral profiles of CaP, indicating changes in the local phosphate environments. The T_1_ relaxation time of the major ^31^P resonance at ∼2.5 ppm decreased from 54 to 39 s upon TA incorporation, while smaller changes were observed for the ^31^P resonance at approximately −1.8 ppm. These changes in T_1_ relaxation behavior further indicate that TA incorporation modifies the local environment and structural heterogeneity around phosphate species. X‐ray diffraction (XRD) analysis was used to investigate crystallinity differences between CaP and CaP–TA particles (Figure 3d). The CaP particles exhibited multiple distinct peaks in the 2*θ* range of 5–70°, characteristic of a crystalline monetite phase (ICDD‐PDF card No. 01‐072‐0713) [47]. However, upon incorporation of TA, these peaks broadened and their intensity decreased, which is indicative of a loss in long‐range order, suggesting a transition toward an amorphous structure in the CaP–TA particles [48]. Moreover, an increase in TA from 0.5% to 15% added during precipitation led to more prominent broad peaks within the 2*θ* range of 5°–15°, providing further evidence of enhanced amorphous character in the CaP–TA particles (Figure 3e). Quantitative analysis of the crystallinity revealed a marked decrease from 67.2 to 36.2% with increasing TA content, confirming that TA incorporation progressively disrupted the crystalline structure of CaP (Figure 3f). The surface changes in crystallinity were further investigated by grazing‐incidence wide‐angle X‐ray scattering (GIWAXS) analysis of the CaP and CaP–TA (15%) particles (Figure 3g). The incorporation of TA into the CaP particles reduced the peak intensities within the range of *q* = 0.5–3.5 Å^−1^, indicating altered crystallinity. This observation was also consistent with the scanning electron microscopy (SEM) analysis and selected area electron diffraction (SAED) data from transmission electron microscopy analysis, with both analyses revealing changes in morphology from globular to sharp, wedge‐like shapes and in the crystal lattice of CaP upon TA incorporation (Figure 3h,i). Together with the crystallinity and lattice changes observed by TEM–SAED, XRD, and GIWAXS, the ^31^P solid‐state MAS NMR results, ^31^P T_1_ relaxation measurements, and the Ca–O coordination identified by XPS and FTIR provide evidence that TA incorporation could regulate both local phosphate environments and long‐range order structure of the mineral.

We hypothesize that the reduced crystallinity of CaP–TA particles enhances nutrient availability in soil by accelerating the release of plant nutrients, such as calcium ions and phosphate, as increased structural disorder facilitates greater dissolution [49]. To test this hypothesis, we quantified the release of P and TA from the CaP and CaP–TA particles after immersion in distilled water and acidified water (pH 4 using citric acid) (Figure 3j–m). The amounts of dissolved P increased with increasing TA incorporation into CaP precipitation in both distilled water and citric acid‐acidified conditions (Figure 3j,k). Compared with CaP, CaP–TA (15%) exhibited up to an 85% increase in P dissolution, suggesting that TA incorporation is an effective strategy to enhance the supply of inorganic nutrients to crops under irrigation conditions. In addition, CaP and CaP–TA precipitated at pH 9 were evaluated, and the release of P was monitored in distilled water (pH 6) and acetate buffer (100 mM, pH 5.1) (Figure S3). The observed results were consistent with the P release achieved through mineral precipitation at pH 10.4 (Figure 3j–m). Because citric acid is a common root exudate [50], this approach also confers responsiveness to rhizosphere conditions. The enhanced P dissolution behavior was also maintained in acidic sodium acetate buffer (100 mM, pH 5.1) and in water containing soil organic matter (i.e., humic acid), demonstrating that the observed release profile is preserved under more soil‐relevant chemical conditions (Figure S6). The amounts of co‐released total phenolic compounds were proportional to the initial TA contents in the CaP–TA particles (Figure 3l,m), indicating that the incorporated TA is released concurrently during particle dissolution. The solution pH after 24 h of CaP and CaP–TA incubation in water or citric acid solution was also measured, as shown in Figure S7. The decrease in solution pH with increasing TA content suggests that co‐released TA may contribute to mineral dissolution through acidification. In addition, TA may chelate dissolved metal ions and suppress their reprecipitation with phosphate. Therefore, the enhanced dissolution likely results from the combined effects of reduced mineral crystallinity and TA‐mediated acidification and metal chelation. The co‐released TA may further benefit plant growth, given its known plant‐stimulatory effects [10, 11, 12].

Lettuce seedlings were cultivated in soil pots (Table S1) to evaluate whether the enhanced dissolution and co‐release of TA from CaP–TA particles contribute to improved plant nutrition [50]. As shown in Figure 4a and Figure S8, cultivation in soil treated with CaP–TA particles resulted in improved lettuce growth, as characterized by larger leaf areas, compared to cultivation in soil treated with CaP particles. These results indicate that incorporating TA into crystalline mineral structures can be an effective strategy to promote crop growth and potentially enhance agricultural productivity. Among the treatments examined, CaP–TA particles synthesized with 3% and 8% TA exhibited nearly two‐fold increases in plant biomass relative to CaP particles (Figure 4b). No statistically significant difference in plant growth was observed between the 3% and 8% TA‐loaded CaP treatments, suggesting that increasing the TA content from 3% to 8% did not provide an additional growth benefit under the conditions studied. Accordingly, the optimal TA loading and fertilizer‐use efficiency under field conditions require further investigation. Plant growth enhancement resulting from the application of TA alone (3% or 8%) was minimal compared with that observed for the application of CaP–TA particles. This limited growth response is likely attributable to the fact that TA does not function as a direct plant nutrient. Because the crops were cultivated without deliberate abiotic or biotic stress, the observed growth enhancement cannot be directly attributed to stress‐related physiological effects of TA; however, TA‐mediated mineral dissolution and changes in the soil microbial community may have contributed to the enhanced crop growth [51, 52]. It should be noted that although TA may influence plant physiology and the native soil microbiome, the substantially greater growth observed for the TA‐incorporated minerals compared with TA‐alone treatment indicates that these effects alone cannot account for the enhanced crop performance. Alternatively, as demonstrated in this study, TA‐induced mineral dissolution and changes in the soil microbial community may have contributed to the observed enhancement in crop growth. Other crops, including pak choi and corn, were also grown in CaP–TA‐treated soil, and similar growth enhancements of up to a 2.5‐fold were observed relative to cultivation conducted in CaP‐treated soil, indicating that our TA incorporation strategy has broad applicability across different crop species (Figure 4c,d and Figure S9). Furthermore, different soil types were used to grow each crop (Table S1), suggesting that the superior performance of the TA‐incorporated fertilizers was independent of soil type.

**FIGURE 4:**
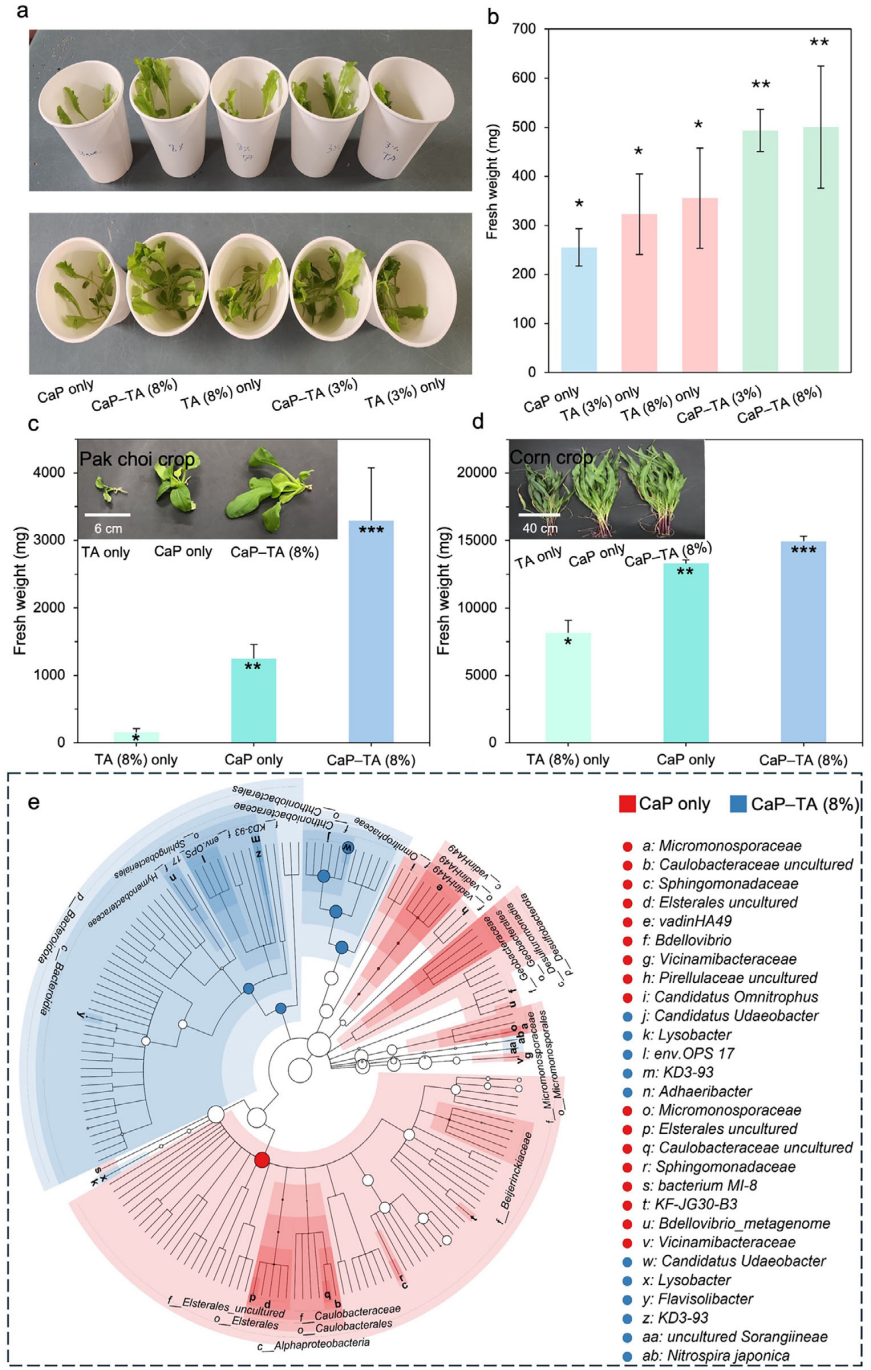
Crop growth studies and soil DNA analysis using CaP and CaP–TA particles. (a) Photographs of lettuces (5 plants per cup) cultivated for 2 weeks in soil pots treated with TA, CaP particles, or CaP–TA particles. (b) Fresh weights of the corresponding lettuces grown in soil pots subjected to the different treatments. The TA only treatments (3% or 8%) contained the same amount of TA as those incorporated in the corresponding CaP–TA (3% or 8%) particles, as determined from TGA of the CaP–TA particles (Figure 2c). (c,d) Fresh weights of pak choi (c) and corn (d) grown for 8 weeks in soil pots treated with TA (8%) only, CaP particles, or CaP–TA (8%) particles. The insets show representative photographs of the aboveground parts of pak choi and corn. The TA only treatment (8%) contained the same amount of TA as that measured in CaP–TA (8%) particles by TGA (Figure 2c). (e) LEfSe analysis (*n* = 4) identifying soil microbial taxa that are differentially abundant in soils treated with CaP and CaP–TA (8%) particles. In (b–d), data are presented as means ± standard deviations (*n* = 5 for (b and c); *n* = 6 for (d)). Different asterisks indicate statistical significance based on one‐way ANOVA with LSD post hoc test (*p* < 0.05).

Soil DNA analyses were performed to profile the microbial community dynamics between the CaP‐ and CaP–TA‐treated soils, as plant polyphenols are known to enrich plant‐beneficial microbes in soil environments [4, 5, 53]. LEfSe (Linear Discriminant Analysis Effect Size) analysis revealed microbial taxa, including *Lysobacter*, *Nitrospira japonica*, and *Adhaeribacter*, that were significantly enriched in soils treated with CaP–TA (Figure 4e and Figure S10). These microbial taxa are associated with plant growth promotion, stress tolerance, and pathogen suppression (Table S2), suggesting that the enhanced lettuce growth observed in CaP–TA‐treated soils can be partially attributed to the enrichment of specific beneficial soil microbes [54]. Because CaP and CaP–TA exhibited different nutrient‐release kinetics, we cannot fully exclude the possibility that, in addition to TA itself, microbial utilization of the released nutrients contributed to the observed differences in the soil microbiome. Further functional analyses, such as metatranscriptomics or metabolomics, can be valuable to evaluate detailed action mechanisms of enriched microbial taxa with CaP–TA.

We extended our approach to struvite, which consists of ammonium, magnesium ions, and phosphate, to assess whether the beneficial effects of TA incorporation are reproducible in other mineral particles containing different metal ions. TGA of the struvite and struvite–TA particles indicate a positive correlation between TA content in the minerals and the amount of TA added during precipitation (Figure 5a,b). The sharp weight loss observed below 200 °C was likely due to the release of ammonia gas from struvite [55]. As observed for the CaP particles, the struvite particles turned brown upon precipitation with TA, which became darker as the amount of TA added during precipitation increased (Figure 5b inset). Elemental analysis revealed that the phosphorus and magnesium contents in the struvite–TA (25%) particles were slightly altered (P:Mg ratio changed from 1.13:1 to 1.04:1) relative to the struvite particles (Figure 5c). This result suggests that the incorporation of TA may disrupt the stoichiometry of the struvite lattice structure. The smaller change in elemental ratio than that observed in the CaP particles could be ascribed to different lattice structures between CaP and struvite or the involvement of additional hydrogen bonding‐based interactions between ammonium and struvite. In addition, the slightly higher recruitment of magnesium with TA is ascribed to TA–magnesium coordination during alkali precipitation. The slower water sedimentation observed in the struvite–TA particles (Figure 5d) was consistent with the behavior seen in the CaP–TA particles, likely due to the presence of hydroxyl groups in the struvite–TA particles. These groups may enhance particle dispersion in water by increasing surface hydration and reducing aggregation, thereby delaying sedimentation. This interpretation is further supported by the total phenolic content in the struvite–TA particles (Figure 5e).

**FIGURE 5:**
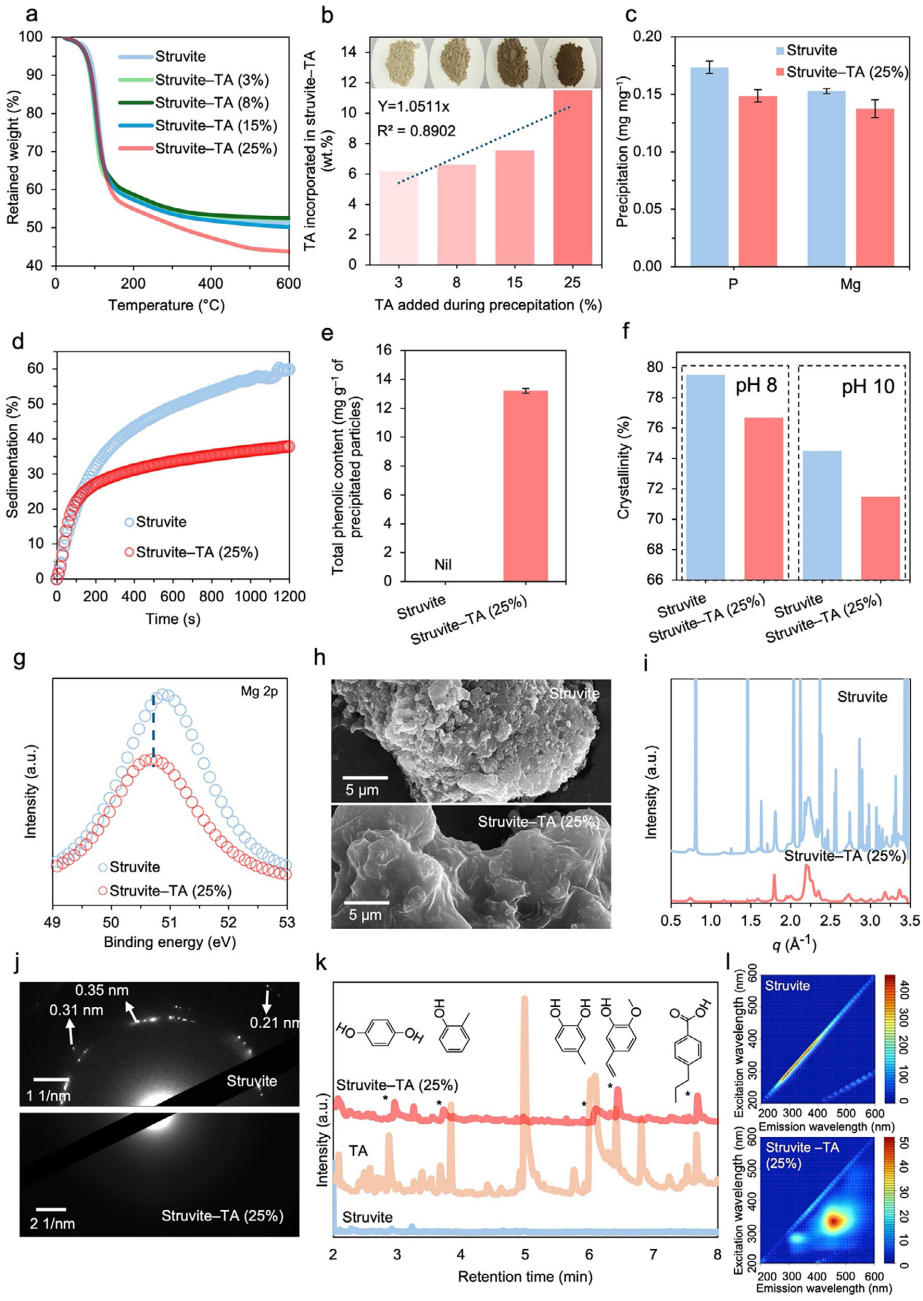
Characterization of TA loading in struvite (struvite–TA particles). (a) TGA curves of struvite and struvite–TA particles with different percentages of incorporated TA. (b) Amount of TA incorporated in struvite–TA particles determined from TGA measurements from 200–600°C in (a). (c) Contents of Mg and P elements in precipitated struvite and struvite–TA (25%) particles measured by inductively coupled plasma atomic emission spectroscopy. (d) Sedimentation kinetics of struvite and struvite–TA (25%) particles. (e) Total phenolic content in struvite and struvite–TA (25%) particles. (f) Crystallinity of struvite and struvite–TA (25%) particles measured from XRD spectra in Figure S11. (g) Mg 2p XPS spectra of struvite and struvite–TA (25%) particles. (h–j) SEM images (h), GIWAXS spectra (i), and SAED patterns (j) of struvite and struvite–TA (25%) particles. (k) Pyrolysis–GC/MS chromatograms of TA, and struvite and struvite–TA (25%) particles. (l) FEEM images of struvite and struvite–TA (25%) particles dissolved in 0.1 N HCl.

We also investigated the effect of TA incorporation on the crystallinity of struvite at different pHs (i.e., 8 and 10) using the alkaline pH‐induced precipitation method (Figure 5f and Figure S11). Under both pH conditions, TA incorporation reduced the crystallinity of the mineral particles. This was evidenced from the reduced intensity of the peaks within 2*θ* ranges of 5°–10°, 25°–35°, and 40°–55° in the XRD patterns of struvite–TA relative to the XRD patterns of struvite; this result suggests that TA coordination with magnesium interferes with crystalline ordering of struvite components. Furthermore, precipitation at a higher pH resulted in the formation of less crystalline struvite particles with TA. Under alkaline conditions, the equilibrium between ammonium (NH_4_^+^) and ammonia (NH_3_) shifts toward the gaseous NH_3_ form, leading to a depletion of available NH_4_^+^ ions in solution. This disturbs the stoichiometric balance required for struvite crystal growth. Simultaneously, the increase in hydroxide concentration promotes the formation of TA–magnesium coordination, which further limits the availability of free Mg^2+^ ions needed for struvite assembly. These conditions accelerate precipitation but prevent the organized incorporation of ions into the orthorhombic struvite lattice, resulting in poorly crystalline materials [56, 57].

The Mg 2p XPS spectrum of the struvite–TA particles exhibited a slight shift to a lower binding energy relative to that of the struvite particles, indicating changes in the Mg electron density through coordination to the oxygen atoms of hydroxyl groups in TA (Figure 5g). Furthermore, the N 1s XPS spectra revealed distinct differences between the struvite and struvite–TA particles (Figure S12). Both the spectra of struvite and struvite–TA particles exhibited characteristic NH_4_^+^ peaks at 402 eV but that of the struvite–TA particles featured an additional peak at approximately 401 eV. This peak was likely due to the formation of a pyridinic nitrogen structure, possibly arising from a Schiff base reaction between ammonia and TA during the incorporation process.

SEM analysis of the struvite–TA particles revealed a morphological transformation toward a smoother surface structure relative to the struvite particles (Figure 5h), indicating successful incorporation of TA into or onto the struvite minerals. Both GIWAXS and SAED analyses demonstrated reduced crystallinity, further confirming that the incorporation of TA induces the amorphization of struvite (Figure 5i,j). Moreover, struvite–TA exhibited changes in the ^31^P solid‐state MAS NMR spectral profile, where the chemical shift and T_1_ values for all ^31^P resonances were influenced through the short‐range structural disorder by incorporation of TA (Figures S13 and S14). It is noted that while TA reduces the crystallinity of both CaP and struvite, the underlying coordination environments may differ because Ca^2+^ and Mg^2+^ possess distinct coordination, hydration characteristics, and ligand exchange kinetics. Resolving these atomistic coordination structures would require advanced structural characterization. Pyrolysis–GC/MS chromatograms of struvite and struvite–TA were consistent with those of CaP and CaP–TA, in that phenolic, catechol, and carboxylate moieties were identified as being involved in coordination with Mg (Figure 5k). The redshift observed in the FEEM image of struvite–TA, with excitation wavelengths of 300–360 nm and emission wavelengths of 400–530 nm, indicates the formation of Mg–TA coordination (Figure 5l). Changes in the dissolution pattern of struvite and struvite–TA in distilled water, citric acid‐acidified water (pH 4), acidic sodium acetate buffer (100 mm, pH 5.1), and water containing soil organic matter (i.e., humic acid) were assessed (Figures S15 and S16). Up to two‐fold increases in P dissolution were observed as a function of the amount of TA incorporated. The release of P from struvite and struvite–TA, precipitated at pH 9, in water or acetate buffer also showed the same trend as that observed for the CaP‐type minerals, where incorporation of TA could increase the dissolution of P (Figure S17). Moreover, the solution pH after 24 h of struvite and struvite–TA incubation in water or citric acid solutions was measured (Figure S18); consistent with the observation in CaP and CaP–TA, increasing TA content led to a decrease in the pH of the solution (i.e., water or citric acid media), thereby indicating the involvement of released TA in particle dissolution. The extent of co‐released TA also increased with increasing TA incorporated amounts (Figure S15), indicating that TA incorporation likely contributes to the co‐delivery of crop nutrients and TA under irrigation and rhizosphere conditions.

An improvement in lettuce growth was also observed upon the application of struvite–TA particles to soils, as shown in Figure 6a,b. Specifically, plants grown in struvite–TA‐containing soils exhibited approximately a 30% increase in biomass weight relative to those grown in struvite‐containing soils. In contrast, the application of TA only in the soil resulted in marginal growth, likely due to its lack of essential plant nutrients such as phosphorus, magnesium, and nitrogen. This result suggests that the enhanced plant nutrition observed is not solely due to TA itself but rather to the effects resulting from its incorporation into the struvite matrix and subsequent faster dissolution kinetics. Likewise with the growth enhancement observed with the application of CaP–TA particles (Figure 4), growth promotion was observed for other crops (pak choi and corn), suggesting that TA incorporation into struvite is broadly applicable across diverse crop species (Figure 6c,d and Figure S19). Changes in the soil microbiome were also observed following the application of struvite–TA, in which *Sphingomonas*, *Lysobacter*, *Massilia*, and *Chitinophagaceae* (i.e., microbial taxa known to promote plant growth, enhance stress tolerance, and suppress pathogens (Table S3)) were significantly enriched (Figure 6e and Figure S20).

**FIGURE 6:**
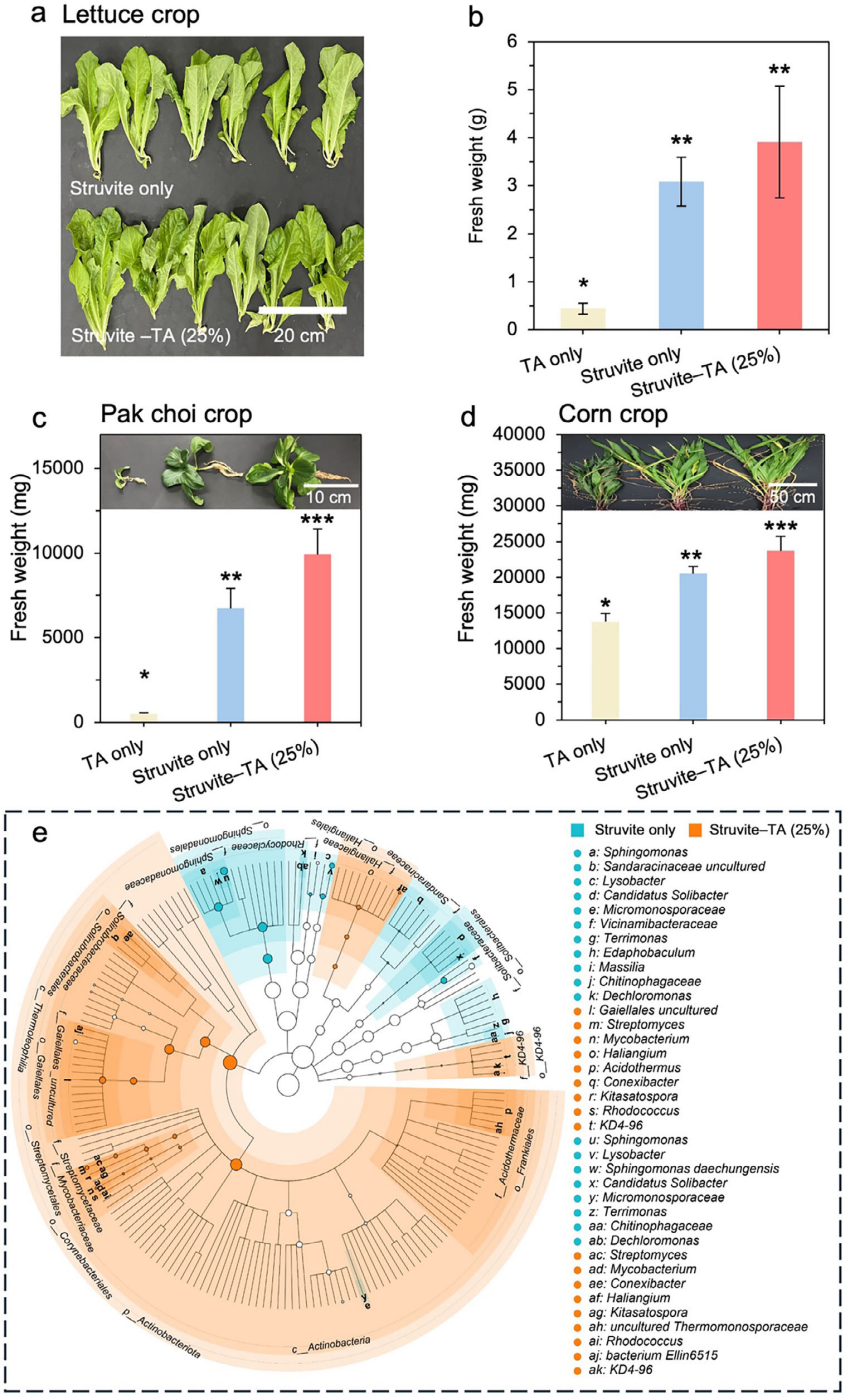
Crop growth studies and soil DNA analysis using struvite and struvite–TA particles. (a,b) Photographs (a) and fresh weights (b) of lettuces cultivated in soil pots containing struvite and struvite–TA (25%) particles for 4 weeks. (c,d) Fresh weights of pak choi (c) and corn (d) cultivated in soil pots containing struvite and struvite–TA (25%) particles for 11 weeks. The insets show representative photographs of the aboveground parts of pak choi and corn. (e) LEfSe analysis (*n* = 4) identifying soil microbial taxa that are differentially abundant in soils treated with struvite and struvite–TA (25%) particles. In (b–d), data are presented as means ± standard deviations (*n* = 6 for (b and d); *n* = 4 for (c)). Different asterisks indicate statistical significance based on one‐way analysis ANOVA with LSD post hoc test (*p* < 0.05).

Understanding the roles of plant secondary metabolites is important for their applications, considering that they can be extracted in bulk from renewable plant sources [58]. These metabolites are naturally produced by plants; however, they have been mainly used in cosmetics and food packaging owing to their known biological effects in humans [59]. Our findings on their natural roles in soils open avenues for applying these compounds in ways that benefit the plants themselves. In addition, mineral particles used in this study (i.e., CaP and struvite) are emerging as alternatives to conventional chemical fertilizers derived from the Haber–Bosch process and phosphate rock, as they can be synthesized via alkali‐induced wet‐precipitation of nitrogen‐ and phosphorus‐containing ions derived from circular economy‐based bioresources [34], such as livestock bones and wastewater activated sludges [35]. Regarding the long‐term stability of the developed fertilizers, our previous study on metal–phenolic networks for developing encapsulated urea fertilizers demonstrated that metal–phenolic networks can undergo dynamic structural evolution during aging, with increased metal–phenolic complexation and associated changes in their physicochemical properties [18]. Thus, aging of TA‐incorporated mineral particles may not necessarily result in recrystallization; continued metal–phenolic coordination and structural reorganization could instead maintain or further increase mineral structural disorder. Nevertheless, whether such behavior occurs for CaP–TA and struvite–TA during long‐term storage remains to be established. In addition to CaP and struvite, minerals containing metals, such as Fe and Al, have potential to be modified based on the polyphenolic complex coordination approach. Our polyphenol‐loaded mineral strategy presents a promising route to developing soil‐compatible and nontoxic alternatives to conventional fertilizers, bridging structural mineralogy with sustainable agriculture.

## 3 Conclusion

This study revealed that plant‐derived polyphenols actively regulate soil fertility by restructuring both short‐ and long‐range structural ordering of mineral matrices to reduce their crystallinity through the incorporation of metal–phenolic complexes. Mineral dissolution and subsequent transformation into TA‐loaded minerals were demonstrated using TA‐driven soil leachates. By translating these natural phenomena into practical fertilizer developments, we engineered TA‐loaded CaP and struvite mineral particles exhibiting accelerated dissolution kinetics, attributable to TA‐driven destabilization of mineral structures. The application of these organic–inorganic hybrid materials led to enhanced crop productivity across multiple species (lettuce, pak choi, and corn), which was realized through synergistic effects of increased nutrient availability (i.e., P and metal ions), codelivery of crop‐stimulatory TA and enrichment of crop‐beneficial microbes. Overall, our findings provide a mechanistic basis for integrating natural plant–soil–microbe feedback processes toward scalable mineral engineering and offer a pathway for improving the performance of fertilizers. The present results demonstrate the potential of TA‐incorporated minerals as fertilizer materials. However, additional factors require further evaluation for large‐scale implementation. These include the cost and sustainable supply of TA, optimization and scale‐up of the coprecipitation process, and the storage stability of the resulting low‐crystallinity minerals. Further assessments of the economic feasibility of the manufacturing process will therefore be required to optimize the amount of TA used for bulk production. Long‐term studies are also needed to determine whether the structural and dissolution characteristics of the TA‐incorporated minerals are maintained during storage.

## Author Contributions

**Jong‐Rok Jeon**: conceptualization, data curation, formal analysis, funding acquisition, investigation, methodology, project administration, resources, software, supervision, validation, visualization, writing – original draft, writing – review and editing. **Omid Mazaheri**: conceptualization, data curation, formal analysis, investigation, methodology, project administration, software, supervision, validation, visualization, writing – original draft, writing – review and editing. **Tianzheng Wang**: data curation, formal analysis, investigation, methodology, validation, writing – review and editing. **Ali Zavabeti**: data curation, formal analysis, investigation, methodology, validation, writing – review and editing. **Ho Young Yoon**: data curation, formal analysis, investigation, methodology, validation, writing – review and editing. **Nguyen Thanh Phong**: data curation, formal analysis, investigation, methodology, validation, writing – review and editing. **Eun‐Nam Joe**: data curation, formal analysis, investigation, methodology, validation, writing – review and editing. **Zhixing Lin**: investigation, methodology, validation, writing – review and editing. **Shuaijun Pan**: formal analysis, investigation, methodology, validation, writing – review and editing. **Chan‐Jin Kim**: data curation, formal analysis, investigation, methodology, validation, writing – review and editing. **Frank Caruso**: conceptualization, funding acquisition, project administration, resources, supervision, validation, visualization, writing – review and editing.

## Conflicts of Interest

F.C., J.‐R.J., and O.M. have filed a patent application (PCT/KR2025/016691) for this technology. The remaining authors declare no competing interest.

## Supporting information




**Supporting File**: advs77994‐sup‐0001‐SuppMat.pdf.

## Data Availability

The data that support the findings of this study are available from the corresponding author. Raw data for the microbial community analyses are available in the NCBI database under accession number PRJNA145464.
