# Supplementary material for: Metal–Phenolic Complexation Governs Soil Mineral Crystallinity to Enhance Crop Growth
Source: Adv Sci (Weinh). 2026 Sep 30:e77994. Online ahead of print. doi: 10.1002/advs.77994 (PMC13627769; doi:10.1002/advs.77994)
Supplement: Supplementary file 1 [file ADVS-9999-e77994-s001.pdf]

Supporting Information

Metal–Phenolic Complexation Governs Soil Mineral Crystallinity to Enhance Crop Growth

Jong-Rok Jeon*, Omid Mazaheri, Tianzheng Wang, Ali Zavabeti, Ho Young Yoon, Nguyen Thanh Phong, Eun-Nam Joe, Zhixing Lin, Shuaijun Pan, Chan-Jin Kim, and Frank Caruso*

J.-R. Jeon, O. Mazaheri, T. Wang, Z. Lin, S. Pan, F. Caruso
Department of Chemical Engineering, The University of Melbourne, Parkville, Victoria 3010, Australia
E-mail: jrjeon@gnu.ac.kr, fcaruso@unimelb.edu.au

O. Mazaheri, F. Caruso
ARC Research Hub for Smart Fertilisers, The University of Melbourne, Parkville, Victoria 3010, Australia
E-mail: fcaruso@unimelb.edu.au

J.-R. Jeon, H. Y. Yoon, N. T. Phong, E-N. Joe
Division of Applied Life Science (BK21Plus), Gyeongsang National University, Jinju 52828, Republic of Korea
E-mail: jrjeon@gnu.ac.kr

J.-R. Jeon
Department of Applied Life Chemistry, Gyeongsang National University, Jinju 52828, Republic of Korea
E-mail: jrjeon@gnu.ac.kr

J.-R. Jeon
Institute of Agriculture and Life Science, Gyeongsang National University, Jinju 52828, Republic of Korea
E-mail: jrjeon@gnu.ac.kr

A. Zavabeti
Department of Chemical and Environmental Engineering, School of Engineering, RMIT University, Melbourne, Victoria 3000, Australia

C.-J Kim
Department of Chemical Engineering, Gyeongsang National University, Jinju 52828, Republic of Korea

MATERIALS AND METHODS

Materials

Potassium hydroxide (KOH, ACS reagent, 98%), potassium dihydrogen phosphate (KH_2PO_4, ACS reagent, 98%), calcium nitrate tetrahydrate ($Ca(NO_3)_2·4H_2O$, ACS reagent, 98%), tannic acid (TA, ACS reagent, 99%), magnesium chloride hexahydrate ($MgCl_2·6H_2O$, ACS reagent, 99%), ammonium chloride (NH_4Cl, ACS reagent, 99.5%), Folin & Ciocalteu's phenol reagent, citric acid (ACS reagent, 99%), and vanadate-molybdate reagent (for phosphate determination) were purchased from Sigma-Aldrich. Sodium dihydrogen phosphate (NaH_2PO_4, Extra pure, 99%), sodium carbonate (Na_2CO_3, Extra pure, 99%), and nitric acid (70%) were obtained from Daejung Chemicals & Metals (South Korea). Lettuce seeds (Great lakes, Mr Fothergill's seeds) were purchased from Bunnings, Australia. Pak choi and corn were obtained from Mirae Seed Co. (Pohang, South Korea) and Jeilseed Co. Ltd. (Jeungpyeong, South Korea), respectively. Gallic acid hydrate (98%) was acquired from Tokyo Chemical Industry (Japan) and concentrated HCl solution (approximately 36 %) was purchased from Samchun Chemicals Co. Ltd. (South Korea). Humic acid (>70 wt.% humic matter) was purchased from Mycsa AG (Texas, USA). Soil information used for soil leachates and pot experiments are detailed in Table S1. High-purity (Milli-Q) water with a resistivity of 18.2 MΩ cm was obtained from an inline Millipore ROs/Origin water purification system. All solutions were freshly prepared for immediate use for each experiment.

Methods

Soil leachate experiments

To obtain soil leachates with TA application, (non-heat-treated) soil (5 g) or heat-treated soil (5 g; 600 °C for 4 h) was thoroughly mixed with TA (50 mg). Distilled water (5 mL) was then added, and the mixture was shaken and incubated on a roller (60 rpm) at room temperature for 2 h. After incubation, the samples were centrifuged (2700 *g* for 10 min), and the supernatants were collected for subsequent analyses. The control experiment was performed without TA addition. Phosphate (P) concentrations in the supernatants were determined using the vanadate-molybdate colorimetric method, with absorbance readings measured at 470 nm. For metal analysis, aliquots (2 mL) of the supernatants were digested with a mixture of 70% HNO_3 and 36% HCl (30 mL, 1:3 v/v) by heating to 200 °C for 5 h. The digested solutions were subsequently diluted with distilled water and analyzed by inductively coupled plasma–optical emission spectroscopy (ICP-OES; Agilent ICP-720 instrument) to quantify calcium (Ca), aluminum (Al), magnesium (Mg), iron (Fe), and manganese (Mn).

For precipitation, (non-heat-treated) soil (50 g) or heat-treated soil (50 g; 600 °C for 4 h) was thoroughly mixed with TA (500 mg). Distilled water (50 mL) was then added, and the mixture was shaken and incubated on a roller (60 rpm) at room temperature for 2 h. After incubation, the samples were centrifuged (2700 *g* for 10 min), and the supernatants were collected. The pH of soil leachates was adjusted to 10 by dropwise addition of 1 N KOH, inducing particle formation. The precipitates were collected by centrifugation (28,300 *g*, 10 min), washed thrice with distilled water, and finally dried at 85 °C for 24 h. The control experiment was performed without TA application. The average yields (n = 3) of the precipitated particles were 1.60 mg (soil only), 3.13 mg (soil-TA), 2.93 mg (heated soil only), and 3.70 mg (heated soil-TA). The dried powders were then dissolved in 0.1 N HCl (1 mL) for further P and TA quantification using the Folin–Ciocalteu assay (see *Elemental and phenolic compositions of mineral particles* below) and the vanadate-molybdate colorimetric method.

Synthesis of CaP and CaP–TA particles
KH_2PO_4 (5.22 g) and $Ca(NO_3)_2 \cdot 4H_2O$ (4.42 g) were dissolved in distilled water (200 mL). A 5 M KOH solution was added dropwise to gradually increase the pH of the calcium- and phosphate-ion-containing solution to 10.4, unless otherwise specified (e.g., 9), while maintaining the temperature at 35 °C under magnetic stirring at 400 rpm for 30 min. The solution pH was further adjusted to 10.4 by dropwise addition of the 5 M KOH solution and then incubated for another 30 min at 35 °C under magnetic stirring at 400 rpm to form CaP particles. The precipitated CaP particles were collected by centrifugation (2700 *g* for 10 min), then washed three times with distilled water using vigorous vortexing. The resulting particles were dried at 250 °C for 2 h to remove residual water.

For the synthesis of CaP–TA particles, TA was added at varying concentrations (41, 248, 661, and 1243 mg, corresponding to 0.5, 3, 8, and 15%, respectively) and dissolved under vigorous vortexing before the addition of the KOH solution. All other procedures were followed as described for the CaP particles.

Synthesis of struvite and struvite–TA particles
$MgCl_2 \cdot 6H_2O$ (1.52 g), NaH_2PO_4 (1.07 g), and NH_4Cl (0.4 g) were dissolved in distilled water (200 mL), followed by the dropwise addition of 1 M NaOH until the pH reached 10.0, unless otherwise specified (e.g., 8 and 9). The solution was maintained at 25 °C under magnetic stirring at 300 rpm for 2 h. At 30-min intervals, the pH level was readjusted to 10.0 by dropwise addition of 1 M NaOH to form struvite particles. The precipitated struvite particles were retrieved by centrifugation (2700 *g* for 10 min), then washed three times with distilled water using vigorous vortexing. Finally, the resulting particles were dried at 40 °C for 2 days.

For the preparation of struvite–TA particles, TA was added at varying concentrations (57, 154, 288, and 480 mg, corresponding to 3, 8, 15, and 25%, respectively) and dissolved under vigorous vortexing before the addition of the NaOH solution. All other procedures were followed as described for the struvite particles.

Soil pot experiments for crop growth
Dried soil (200 g) was placed into pots (see the details of soil properties in Table S1), and the soil surface was treated with either unmodified CaP mineral particles (70 mg) or TA-modified CaP particles (CaP–TA, 70 mg). For treatments using TA alone, the TA dosage was calculated to match the TA content incorporated in the corresponding CaP–TA particles, as determined by thermogravimetric analysis. Lettuce seeds were sown in commercial bedding soil, and after germination, seedlings were transplanted into the treated soil pots. The pots were incubated in a greenhouse maintained at 23 °C under a 16 h light/8 h dark photoperiod and watered for 2 weeks. The lettuces were then harvested, photographed and weighed to determine their fresh biomass. The same amount of water was applied to all groups.

Lettuce cultivation in soil pots treated with struvite or struvite–TA particles followed the same procedure as described for CaP-based treatments. In these experiments, each pot contained 500 g of dried soil and 150 mg of mineral particles, and the plants were cultivated for 4 weeks.

Pak choi and corn were also cultivated for 8–11 weeks as described above using 500 g of dried soils and 150 mg of mineral particles.

Microbial community analysis

Soils collected from pots at crop harvest were stored at –75 °C before analysis. Soil DNA was extracted from soil samples (500 mg) using the FastDNA SPIN Kit for Soil (MP Biomedicals) according to the manufacturer's instructions. Next-generation sequencing was performed using an iSeq 100 sequencer (Illumina, USA), wherein bacterial community composition was characterized by sequencing the V3–V4 region of the 16S rRNA gene. Amplicons targeting the bacterial 16S rRNA gene were generated using the primer pair 518F (5′-CCAGCAGCCGCGGTAATACG-3′) and 805R (5′-GACTACCAGGGTATCTAATCC-3′). For polymerase chain reaction, purified DNA was initially denatured at 95 °C for 3 min, followed by 27 cycles of amplification at 95 °C for 30 s, 55 °C for 30 s, and 72 °C for 30 s, with a final extension at 72 °C for 5 min. PCR amplicons were purified using Agencourt AMPure XP beads. Index PCR and subsequent purification were performed using the Nextera XT Index Kit (Illumina) and the same bead-based purification protocol. Sequencing libraries were prepared at a final concentration of 80 pM and sequenced with the addition of 60 pM PhiX Sequencing Control v3 (Illumina) as an internal control. Amplicon sequence variants (ASVs) were generated from FASTQ files using QIIME2 (version 2021.2) with the DADA2 pipeline. Taxonomic assignment was performed using the SILVA database (version 138). The Linear Discriminant Analysis Effect Size (LEfSe) analysis using the microbiomeMarker package (ver. 1.13.2) within the R environment was performed across all taxonomic ranks, initially filtering features using the Kruskal–Wallis test ($p < 0.05$) followed by the pairwise Wilcoxon test ($p < 0.05$). Final microbial taxa were selected based on a linear discriminant analysis (LDA) score threshold. Specifically, the default LDA score cutoff of 3.0 was applied for comparison of the mineral particles and TA-incorporated mineral particles.

Kinetics of mineral particle sedimentation

CaP, struvite, CaP–TA, or struvite–TA (25 mg) particles were dispersed in distilled water (3 mL) and ultrasonicated for 4 min. The resulting suspension was then diluted 1:3 (300%) with distilled water. Sedimentation kinetics were monitored by measuring the visible absorbance at 600 nm every 10 s. To define 100% sedimentation, the absorbance of the supernatant obtained after centrifugation at 28,300 *g* for 10 min was used as the baseline.

Elemental and phenolic compositions of mineral particles

For elemental composition analysis of the mineral particles, CaP, CaP–TA, struvite, and struvite–TA (10 mg) particles were digested with a mixture of 70% HNO_3 and 36% HCl (10 mL, 1:3 v/v) by heating to 200 °C for 5 h. The digested solutions were subsequently diluted with distilled water and analyzed by ICP-OES (Agilent ICP-720 instrument) to measure P, Mg, and Ca contents in the mineral particles. The particles (10 mg) were dissolved in 0.1 N HCl solution (1 mL), and aliquots (0.2 mL) of the solution were then mixed with Folin & Ciocalteu's phenol reagent (0.25 mL) and distilled water (2.5 mL). After incubating the mixtures at 23 °C for 5 min, sodium carbonate solution (2.5 mL; 7% Na_2CO_3) was added, followed by an additional 90-min incubation at room temperature. Finally, the absorbance at 550 nm was recorded. Gallic acid was used as a standard for relative quantification.

Dissolution of mineral particles

To assess the solubilization behavior of the mineral particles, each particle sample (10 mg) was dispersed in either distilled water (10 mL), citric acid-acidified water (10 mL, pH 4), 100 mM sodium acetate buffer (10 mL, pH 5.1), or humic acid-containing water (10 mL, 250 ppm). The humic acid suspension in distilled water (0.5 g mL^{-1}) was centrifuged at 19,500 *g* for 10 min, and the resulting supernatant was used to prepare the humic acid-containing water for mineral dissolution. The humic acid content in the supernatant was determined gravimetrically after drying. The suspensions were placed on a roller shaker (60 rpm) and

incubated at room temperature for 24 h. After incubation, the suspensions were centrifuged at 28,300 *g* for 10 min, and the supernatants were collected for analysis. Soluble phosphorus concentrations were determined using the vanadate-molybdate method. Briefly, aliquots (250 μL) of the supernatants were mixed with distilled water (500 μL) and vanadate-molybdate reagent (250 μL). After thorough mixing and incubation for 10 min at room temperature, absorbance was measured at 470 nm. Co-released TA in the supernatants was quantified using the Folin–Ciocalteu assay (see *Elemental and phenolic compositions of mineral particles* above).

<u>Characterization</u>

Fluorescence excitation–emission matrix (FEEM) analysis

FEEM spectra were recorded on a fluorescence spectrophotometer (F-7100, Hitachi, Japan). For soil leachate analysis, aliquots (100 μL) of the supernatants were diluted with distilled water (900 μL) and filtered through a 0.2 μm syringe filter prior to measurement. Emission spectra were recorded over the range of 200–600 nm at 5 nm intervals, with excitation wavelengths ranging from 200–600 nm. The scanning speed was set to 2400 nm min^{-1}, and the response time was 0.5 s. TA dissolved in distilled water (2.5 mg mL^{-1}) was used for comparison.

For mineral particles analysis, the particles (1 mg mL^{-1}) were dissolved in 0.1 N HCl and filtered through a 0.2 μm syringe filter prior to measurement. The filtrate (200 μL) was diluted with distilled water (800 μL), and the diluted sample was analyzed, as described above.

Pyrolysis–gas chromatography–mass spectrometry (GC/MS)

Pyrolysis–GC/MS analysis was conducted by heating samples (2 mg) to 500 °C for 1 min. A pyrolysis–GC/MS system (GCMS-QP2020 NX, Shimadzu) equipped with a DB-5 column (30 m × 0.25 mm, film thickness 0.25 μm, Agilent) was used with helium as the carrier gas at a flow rate of 1.5 mL min^{-1}. The GC oven temperature was programmed from 50 to 100 °C at 30 °C min^{-1} and then from 100 to 300 °C at 10 °C min^{-1}. Injector and transfer line temperatures were set at 250 °C and 280 °C, respectively. Mass spectra were recorded in scan mode (40–700 *m/z*) using electron ionization at 70 eV. Compound identification was performed by comparison with the NIST mass spectral library.

X-ray photoelectron spectroscopy

X-ray photoelectron spectroscopy data were collected on a VG ESCALAB 220i-XL spectrometer using a hemispherical analyzer and monochromatic Al Kα X-ray (1486.6 eV) at 220 W. Pass energies were 100 and 50 eV for survey and high-resolution scans, respectively. Survey scans were recorded with a step size of 1 eV and a dwell time of 10 ms, whereas high-resolution scans were recorded over a 20 eV binding energy range with a step size of 0.05 eV and a dwell time of 100 ms. The base pressure in the analysis chamber was $<8.0 \times 10^{-9}$ mbar, and a low-energy flood gun was used to mitigate surface charging. CasaXPS software was used for data processing and energy calibration, referencing the C 1s peak at 285.0 eV.

X-ray diffraction

X-ray diffraction analysis was conducted on a Bruker D8 Advance X-ray diffractometer using Ni-filtered Cu Kα radiation (1.54 Å, 40 mA, and 40 kV) and a step size of 0.02. No specific preparation methods were used for X-ray diffraction analysis of the minerals. The crystallinity was quantitatively determined using DIFFRAC.EVA software (Version 7.3) by calculating the ratio of the integrated area of the crystalline diffraction peaks to the total integrated area of the

X-ray diffractogram pattern, including both crystalline and amorphous contributions. The corresponding crystallinity values were obtained directly from the software.

Solid-state 31P magic-angle spinning (MAS) NMR spectroscopy
Solid-state ^{31}P MAS NMR spectra were acquired at 25 kHz spinning rate and 288 K on a 600 MHz Oxford spectrometer equipped with an AVANCE NEO console (Bruker, Wissembourg, France) and a 1.9 mm MAS VTN 1H/BB probe. Samples were fitted into 1.9 mm ZrO_2 MAS rotors sealed with torlon drive caps. Chemical shifts were externally referenced to H_3PO_4 (^{31}P carbon at 0 ppm). A 3 μs 90° pulse was used with a 25 kHz CW decoupling during acquisition, and a 250 s recycle delay (set as ~5 × T_1). The spectral window was set to 125 kHz, 4k points were acquired and 64 scans were collected and processed with 10 Hz exponential line broadening and zero filling to 16k points.

Scanning electron microscopy
The morphology of the minerals was examined using a Hitachi FlexSEM 1000 instrument at 15 kV. The surface of the samples was coated with a 10 nm gold layer using an ionization chamber sputter coater (Quorum K575X).

Transmission electron microscopy
Transmission electron microscopy was performed on a JEOL 2100F microscope equipped with an Oxford X-Maxn 5 80T EDX detector and a Gatan OneView 4k camera. An acceleration voltage of 200 kV was used for imaging and mapping. Prior to analysis, the samples were first dispersed in ethanol (0.05 wt% solution), and then a single droplet was dropped onto a formvar carbon-coated copper transmission electron microscopy grid and then dried overnight at room temperature in a vacuum desiccator.

Thermogravimetric analysis
Thermogravimetric analysis was performed on a NETZSCH TG 209 F1 Libra analyzer. Samples were heated from 30 to 600 °C at a rate of 5 °C min^{-1} in an O_2/N_2 (80/20%) stream at 20 mL min^{-1}. Samples were dried overnight at 40 °C prior to thermogravimetric analysis.

Attenuated total reflectance Fourier transform infrared spectroscopy
Attenuated total reflectance Fourier transform infrared spectroscopy was performed on a Bruker TENSOR II system over the range of 400–4000 cm^{-1}, with 64 scans recorded per run.

General instrumentation
Solution pH was measured using a portable LAQUAtwin pH meter (Horiba). The pH meter was calibrated weekly with standard solutions (pH 4, 7, and 9). Mass measurements were performed using a highly accurate (±10 μg) analytical balance (Mettler Toledo XS205).

Statistical analysis
All replicate experiments were conducted independently, and the results are presented as mean ± standard deviation (*n* is defined in the caption of each figure). For the crop experiments, statistical significance was evaluated using one-way analysis of variance (ANOVA), followed by Fisher's least significant difference (LSD) post hoc test. All statistical analyses were performed using Microsoft Excel.

Figures

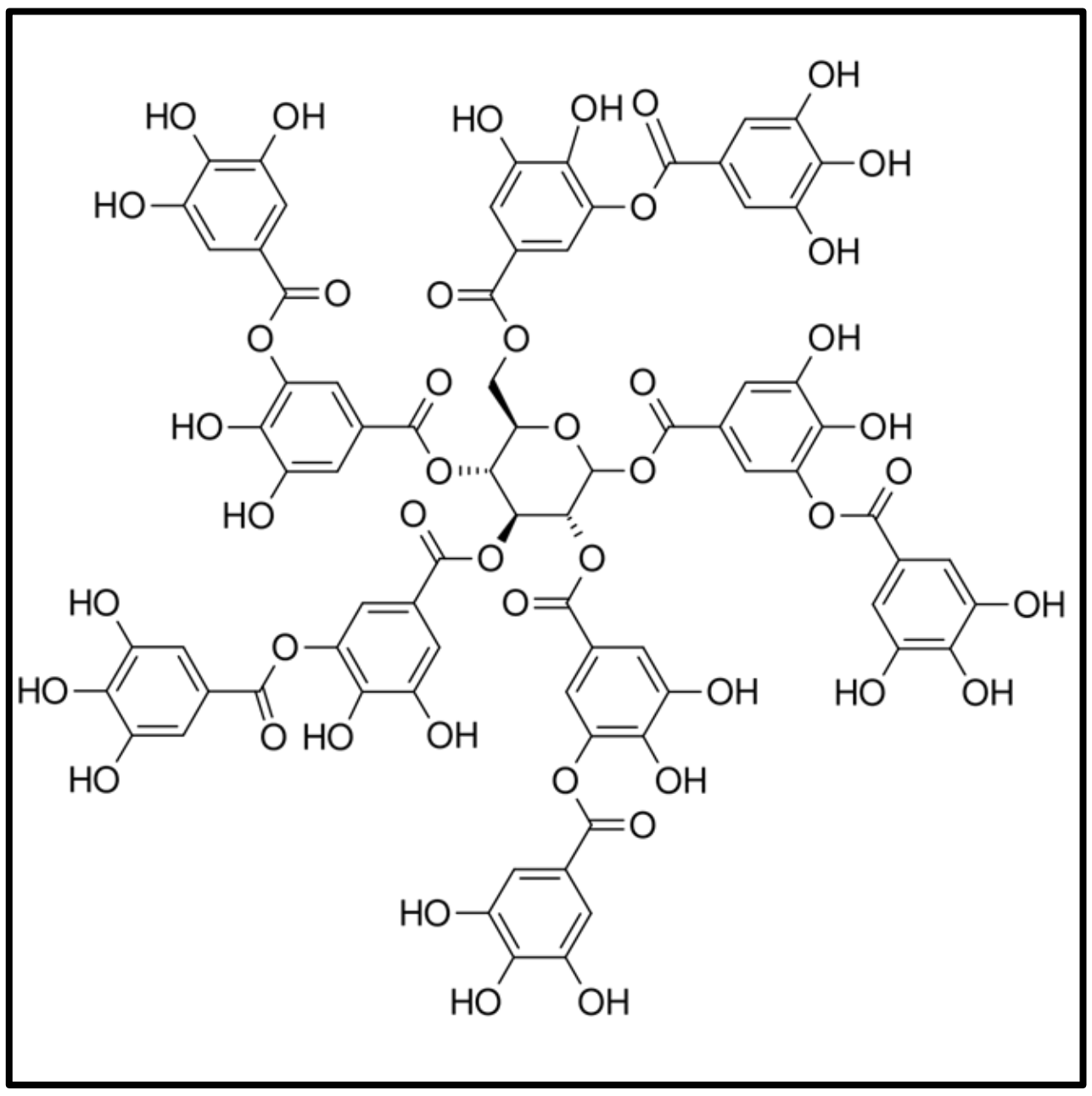

Figure S1. Chemical structure of TA. Although the chemical structure of TA is typically represented as a decagalloyl glucose ($C_{76}H_{52}O_{46}$), it can be a mixture of polygalloyl glucose molecules with different degrees of esterification.

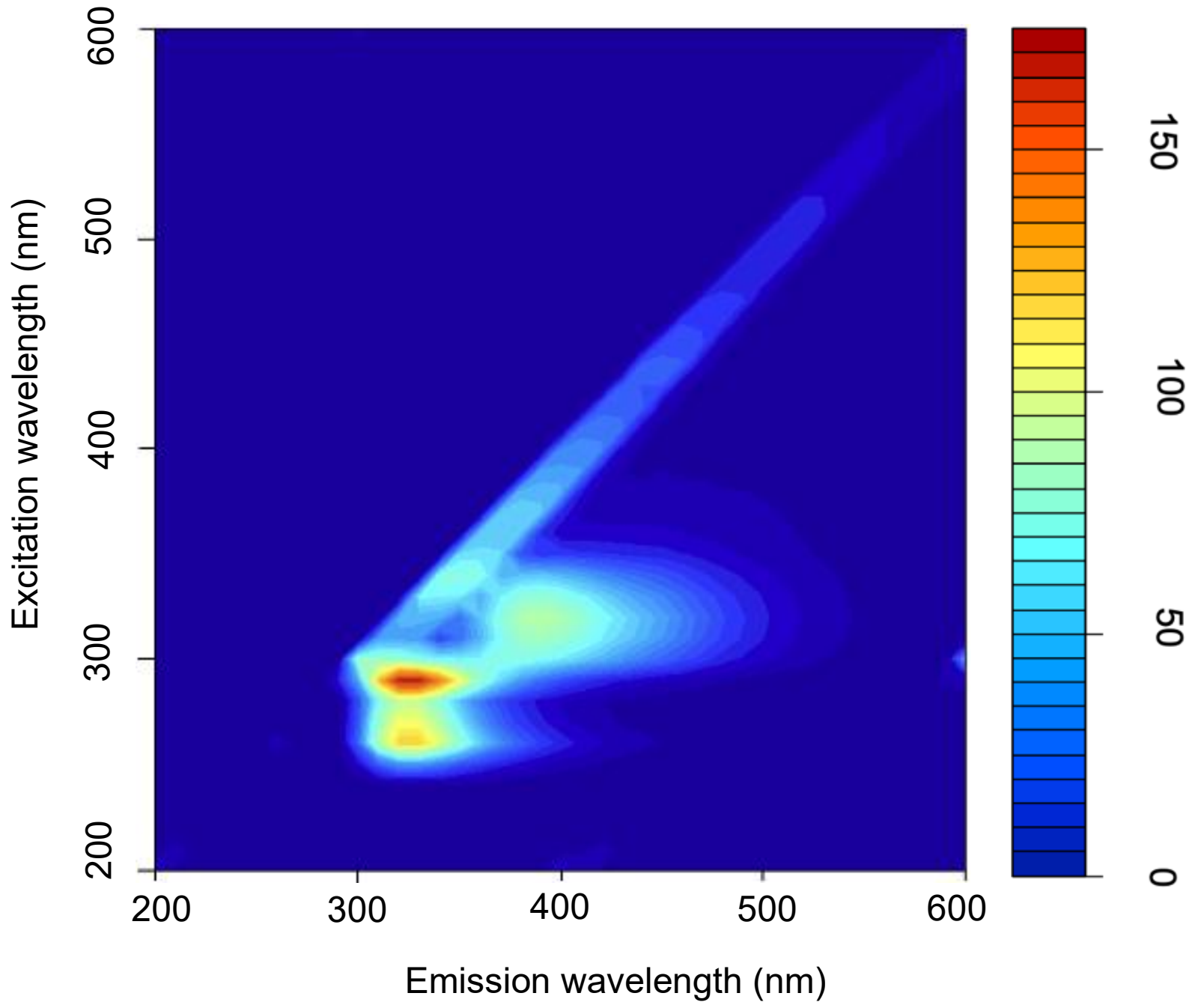

Figure S2. FEEM imaging of TA dissolved in distilled water.

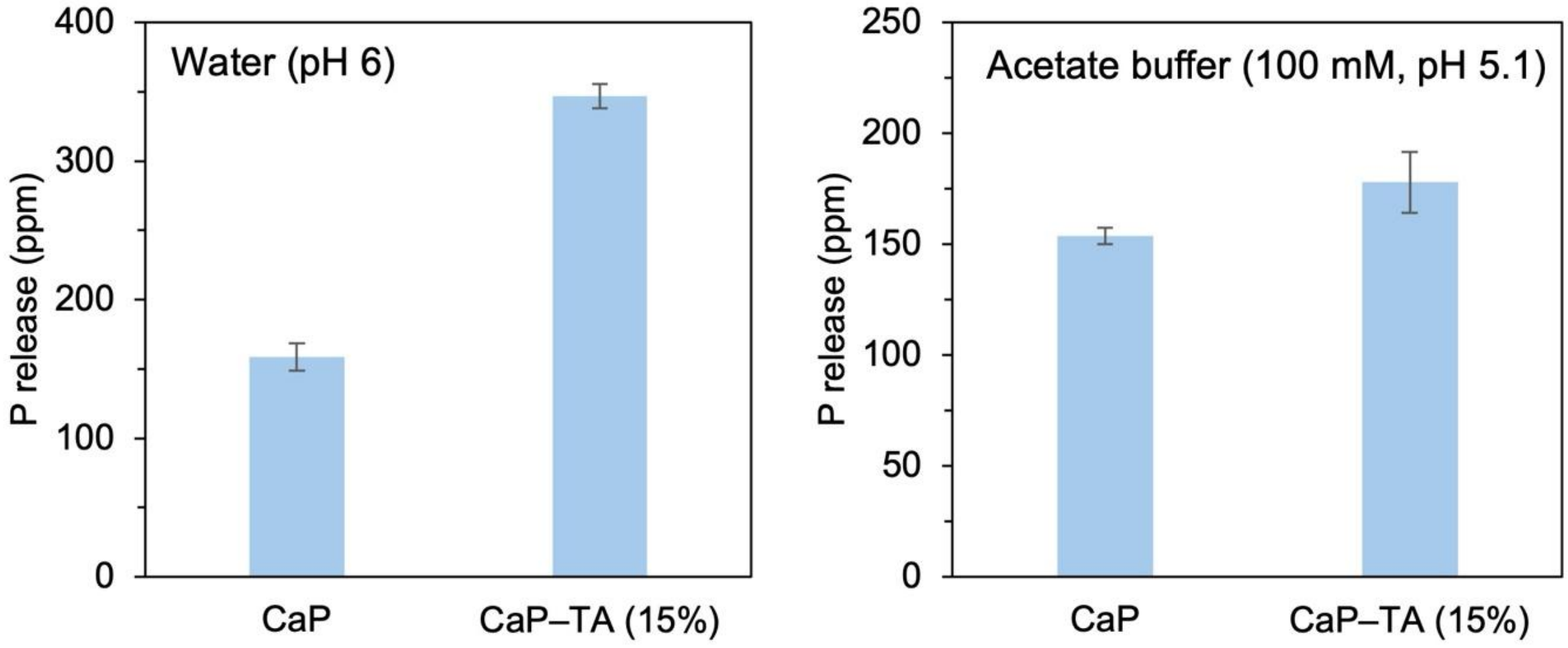

Figure S3. Release of P from CaP and CaP–TA, precipitated at pH 9, in water (pH 6) and acetate buffer media (pH 5.1) after 24 h incubation. Data are presented as mean ± standard deviation (n = 3).

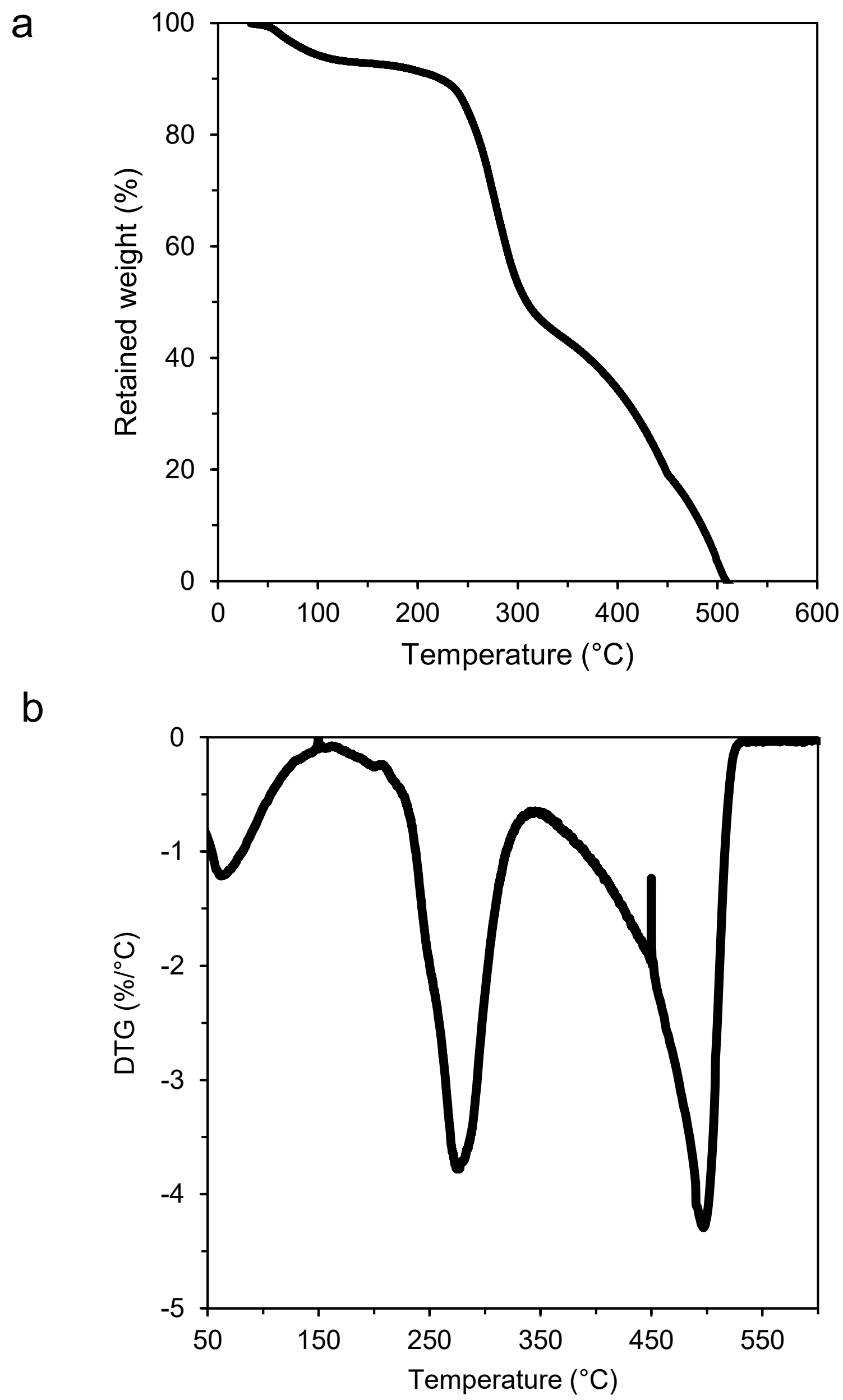

Figure S4. a,b, Thermogravimetric analysis (**a**) and derivative thermogravimetric analysis (**b**) of TA.

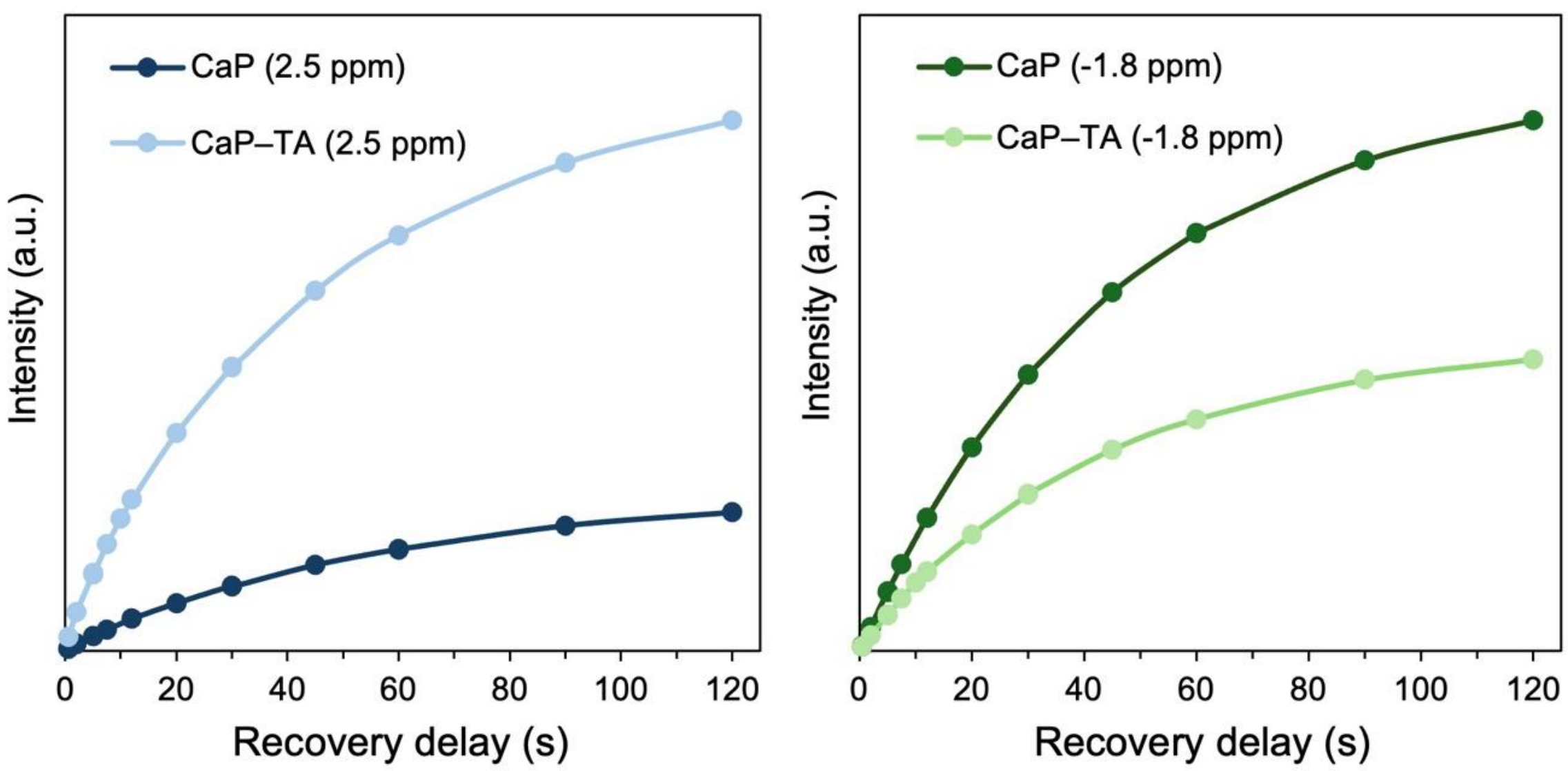

Figure S5. ^{31}P saturation-recovery solid-state MAS NMR measurements of CaP and CaP–TA for two resolved ^{31}P resonances at 2.5 and −1.8 ppm.

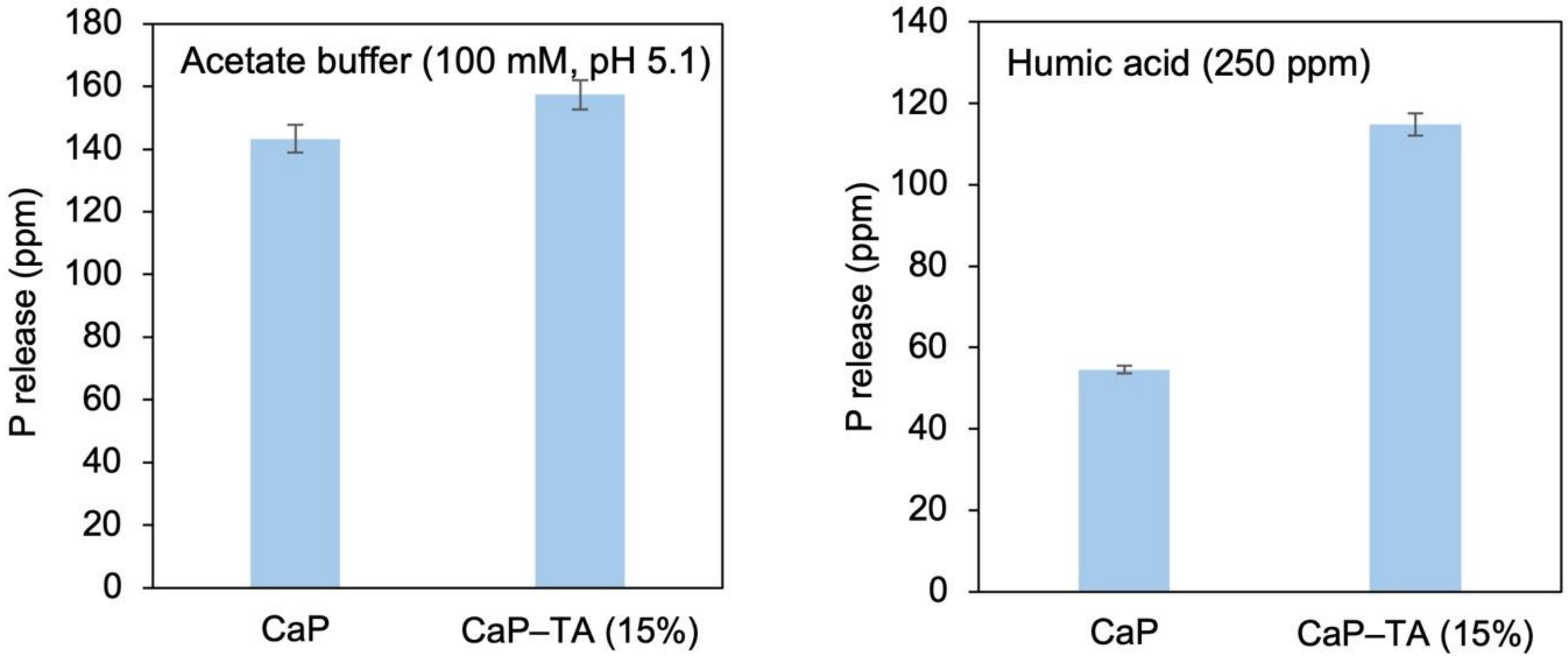

Figure S6. Release of P from CaP and CaP–TA after 24 h incubation in acetate buffer (100 mM, pH 5.1) and humic acid (250 ppm) solutions. Data are presented as mean ± standard deviation (*n* = 3).

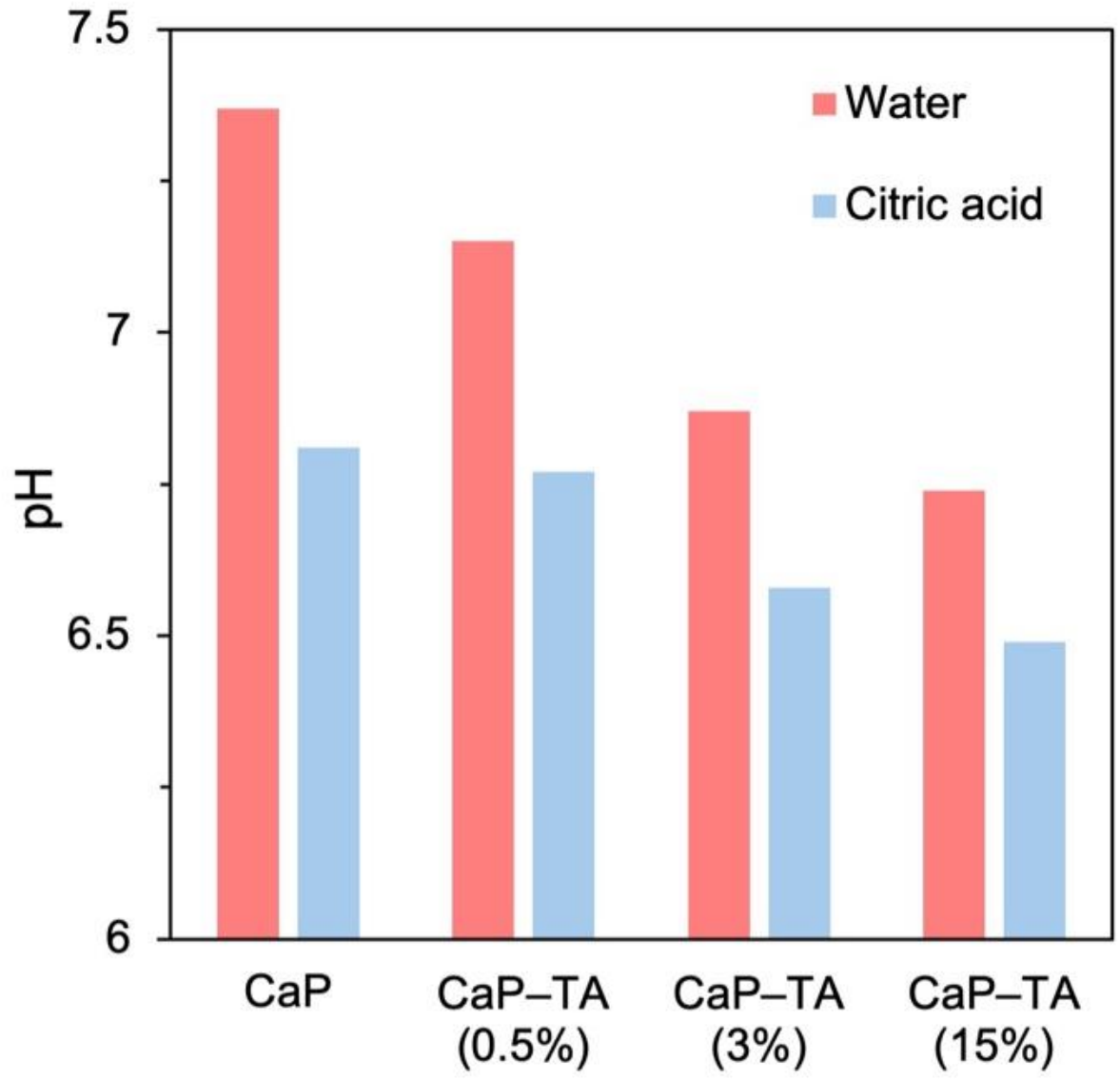

Figure S7. pH values of water and citric acid solutions after incubation of CaP and CaP–TA for 24 h.

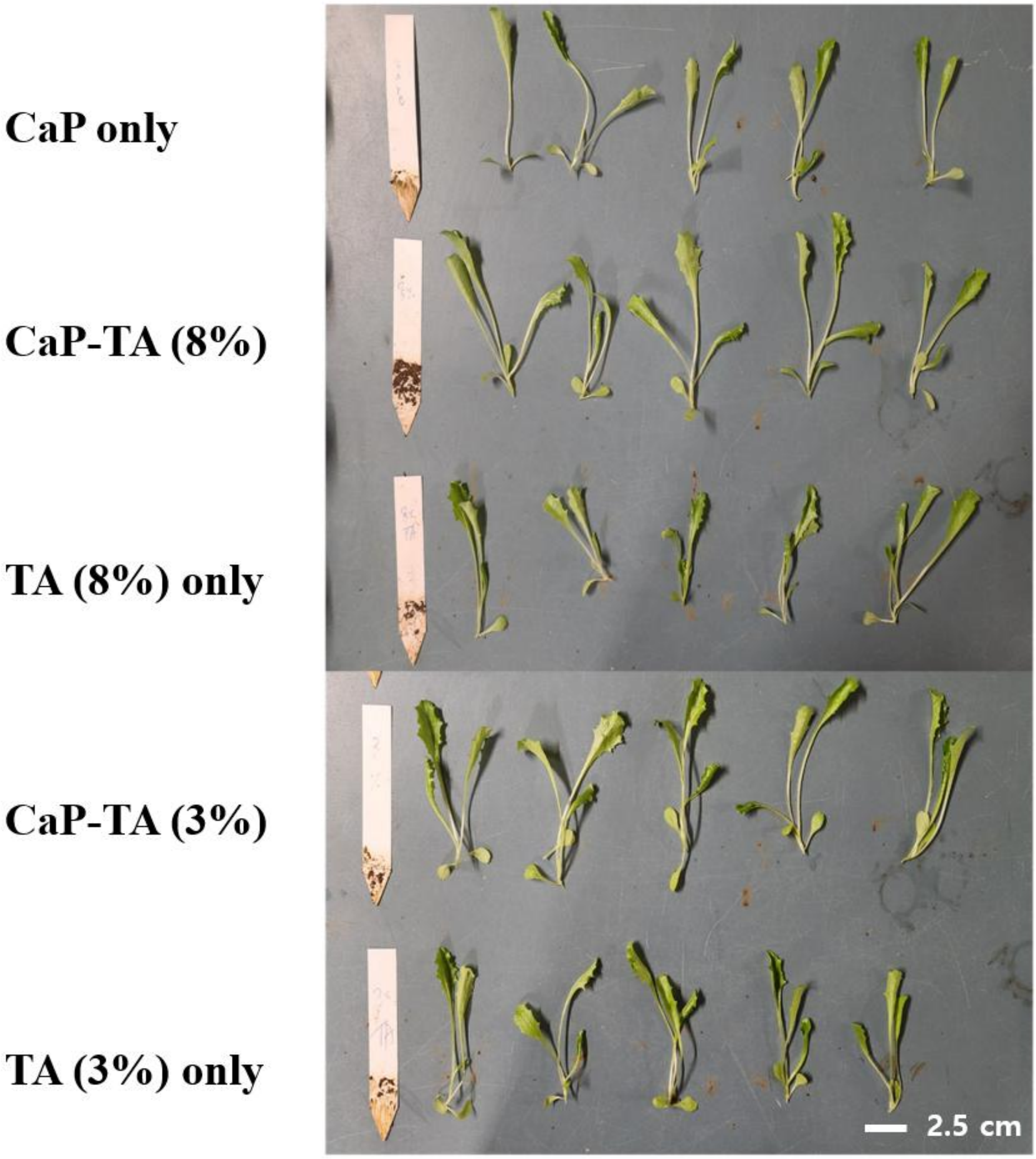

Figure S8. Photographs of the individual lettuce plants shown in Figure 4a. Lettuce cultivation was performed in soil pots treated with TA only or different mineral particles (CaP or CaP–TA).

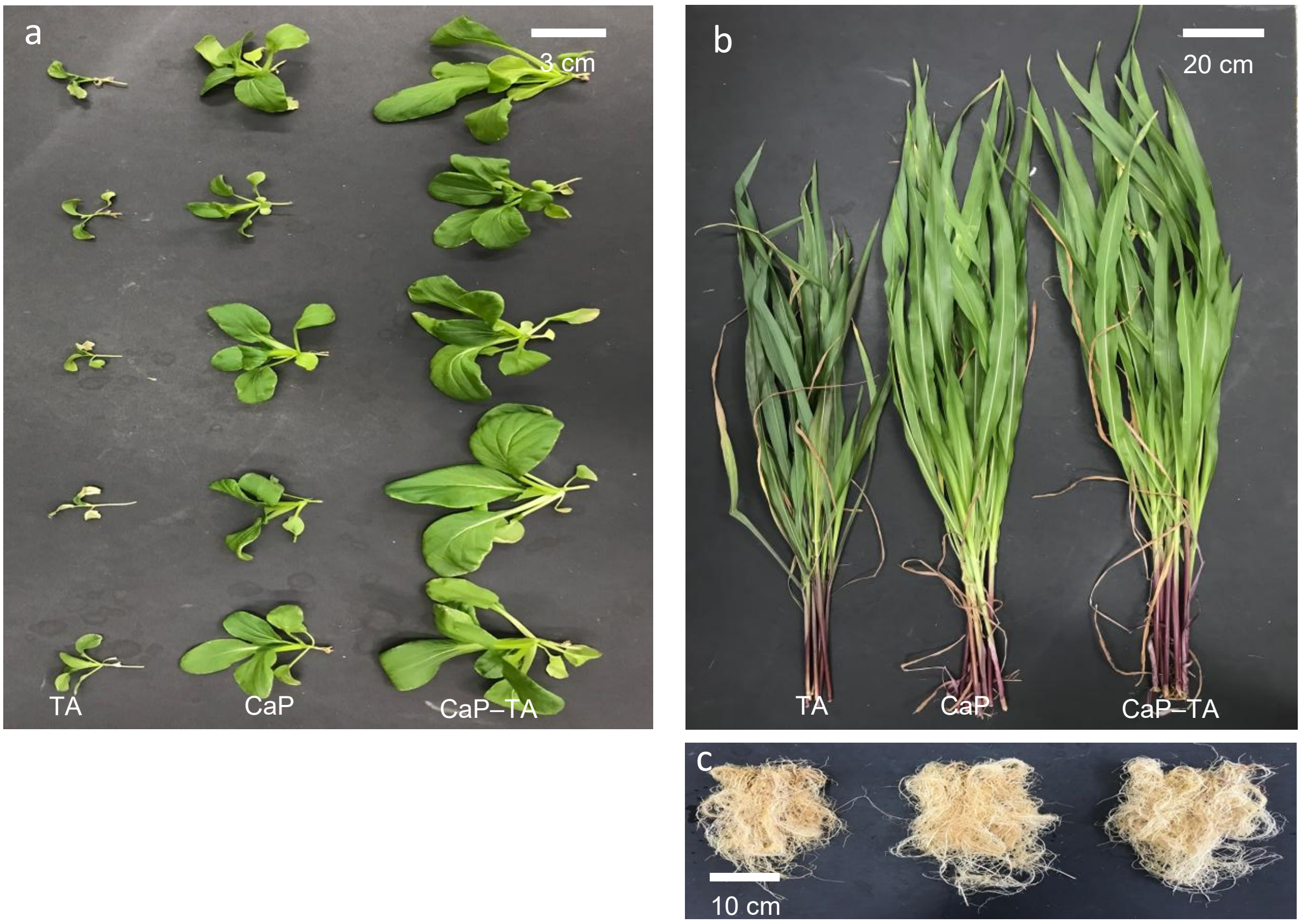

Figure S9. a–c, Photographs of the aboveground parts of pak choi (**a**) and corn (**b**), and corn roots (**c**) grown in soil treated with TA (8%), CaP particles, or CaP–TA (8%) particles.

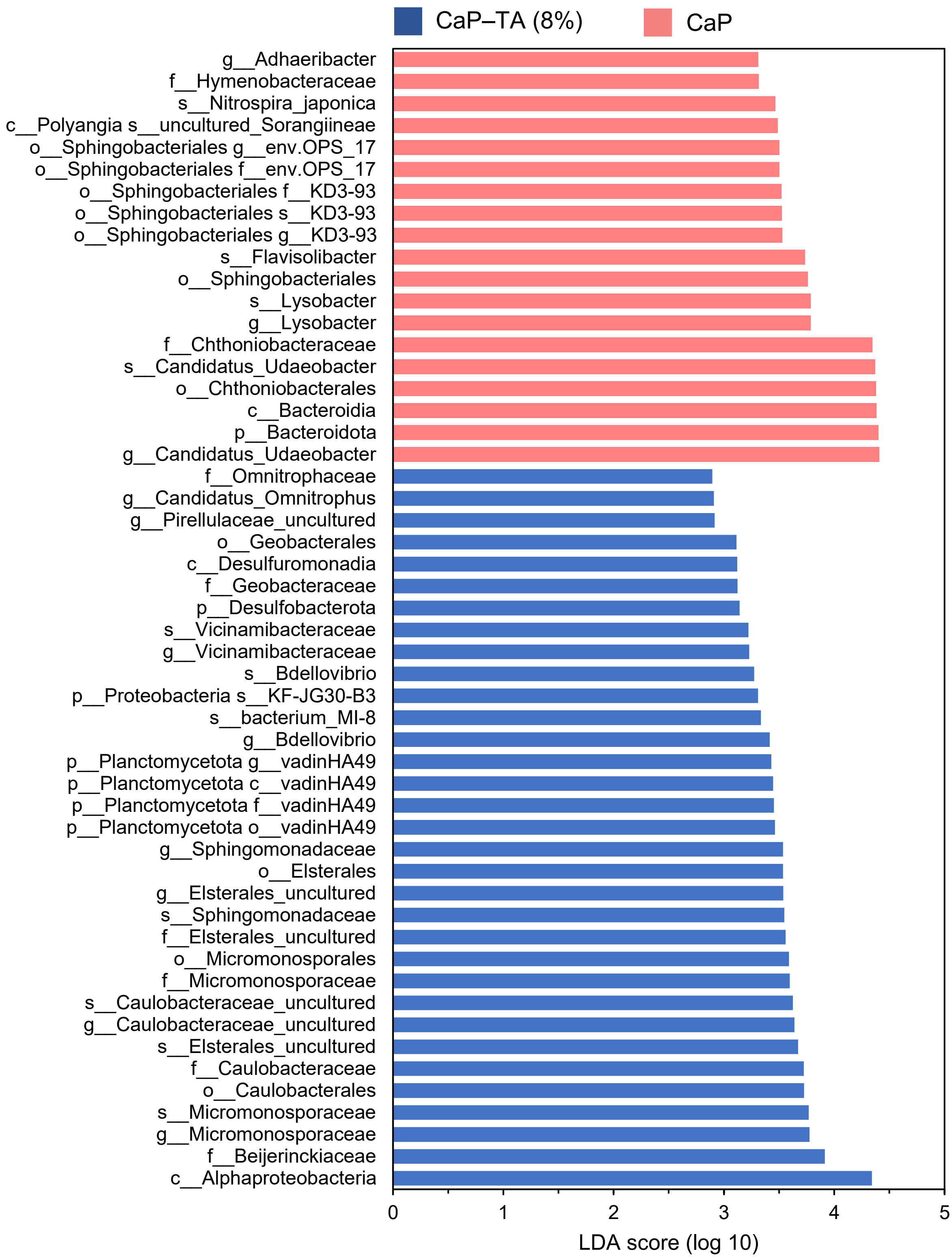

Figure S10. LEfSe analysis (n = 4) identifying soil microbial taxa that are differentially abundant in soils treated with CaP or CaP–TA (8%) particles.

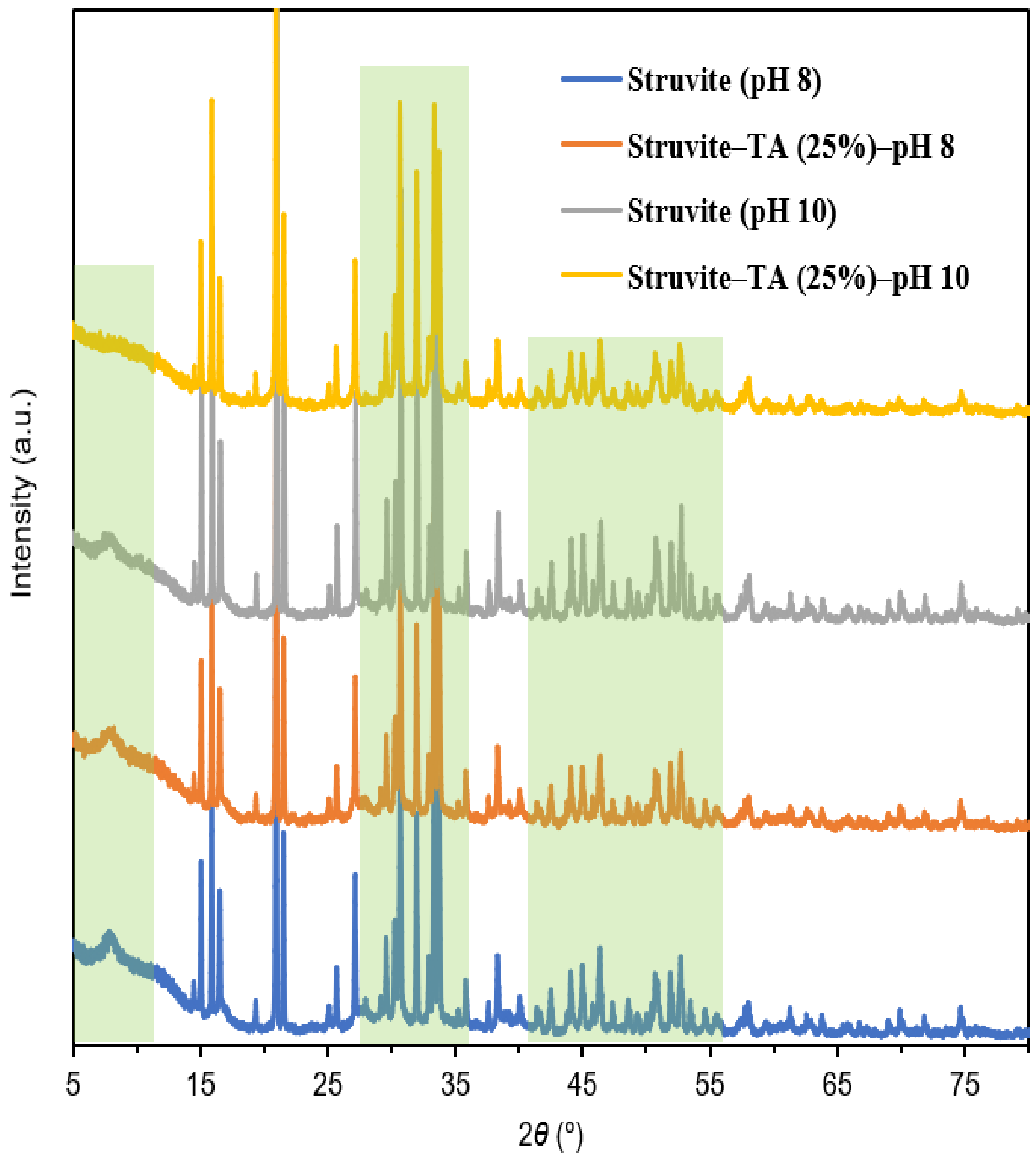

Figure S11. X-ray diffraction patterns of struvite and struvite–TA (25%) particles precipitated at different pHs. The green boxes highlight the reduced peak intensities of struvite–TA (25%) relative to struvite.

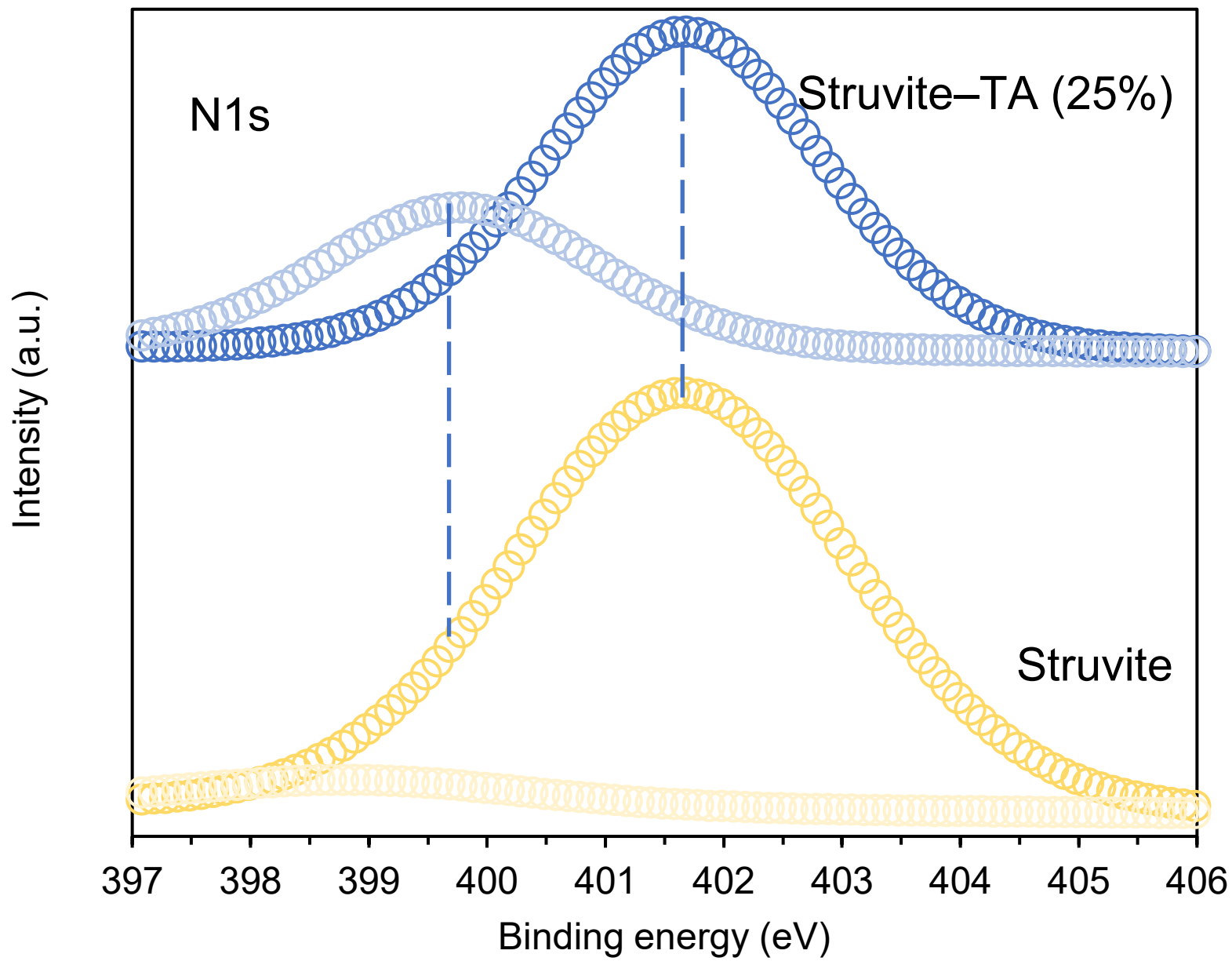

Figure S12. N 1s X-ray photoelectron spectroscopy spectra of struvite and struvite–TA (25%) particles.

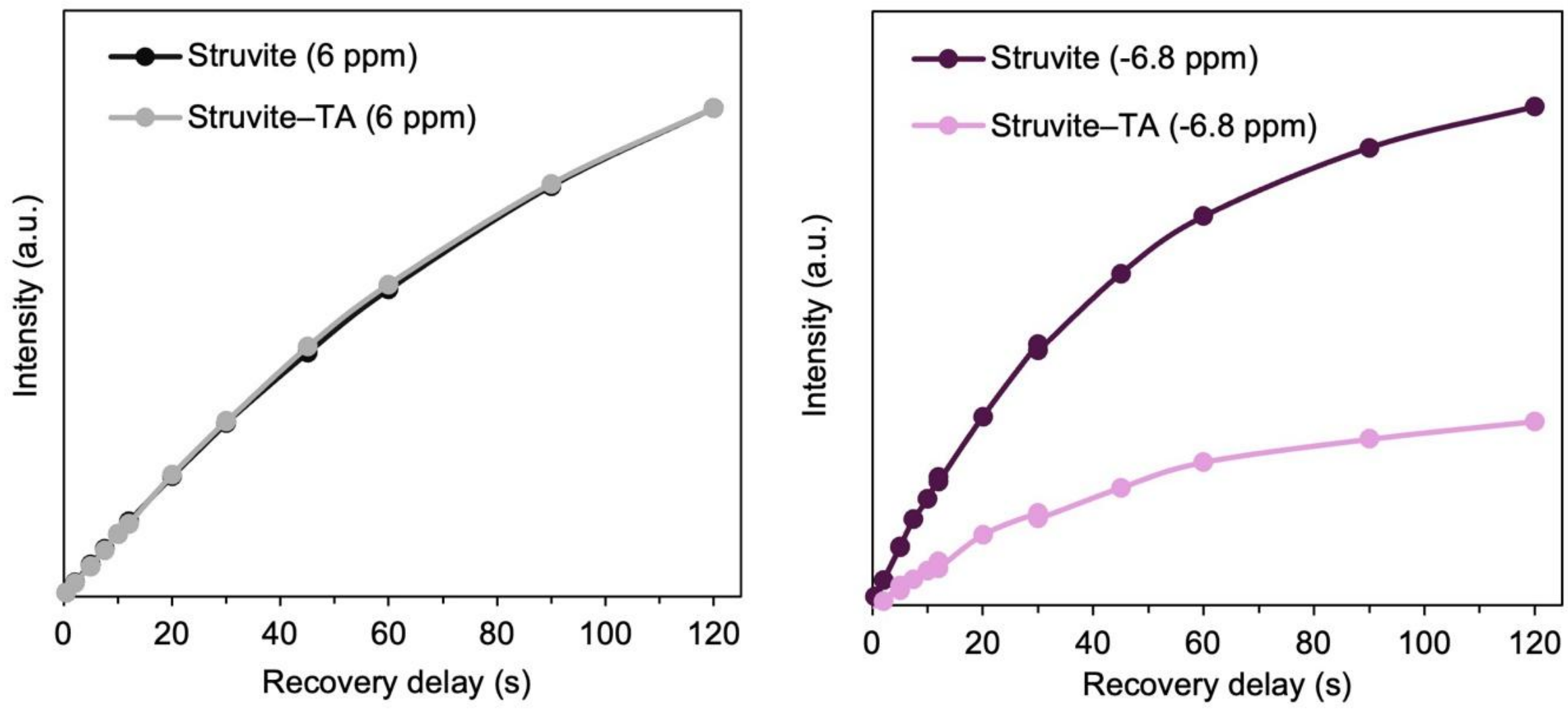

Figure S13. ^{31}P saturation-recovery solid-state MAS NMR measurements of struvite and struvite–TA (25%) for two resolved ^{31}P resonances at 6 and −6.8 ppm.

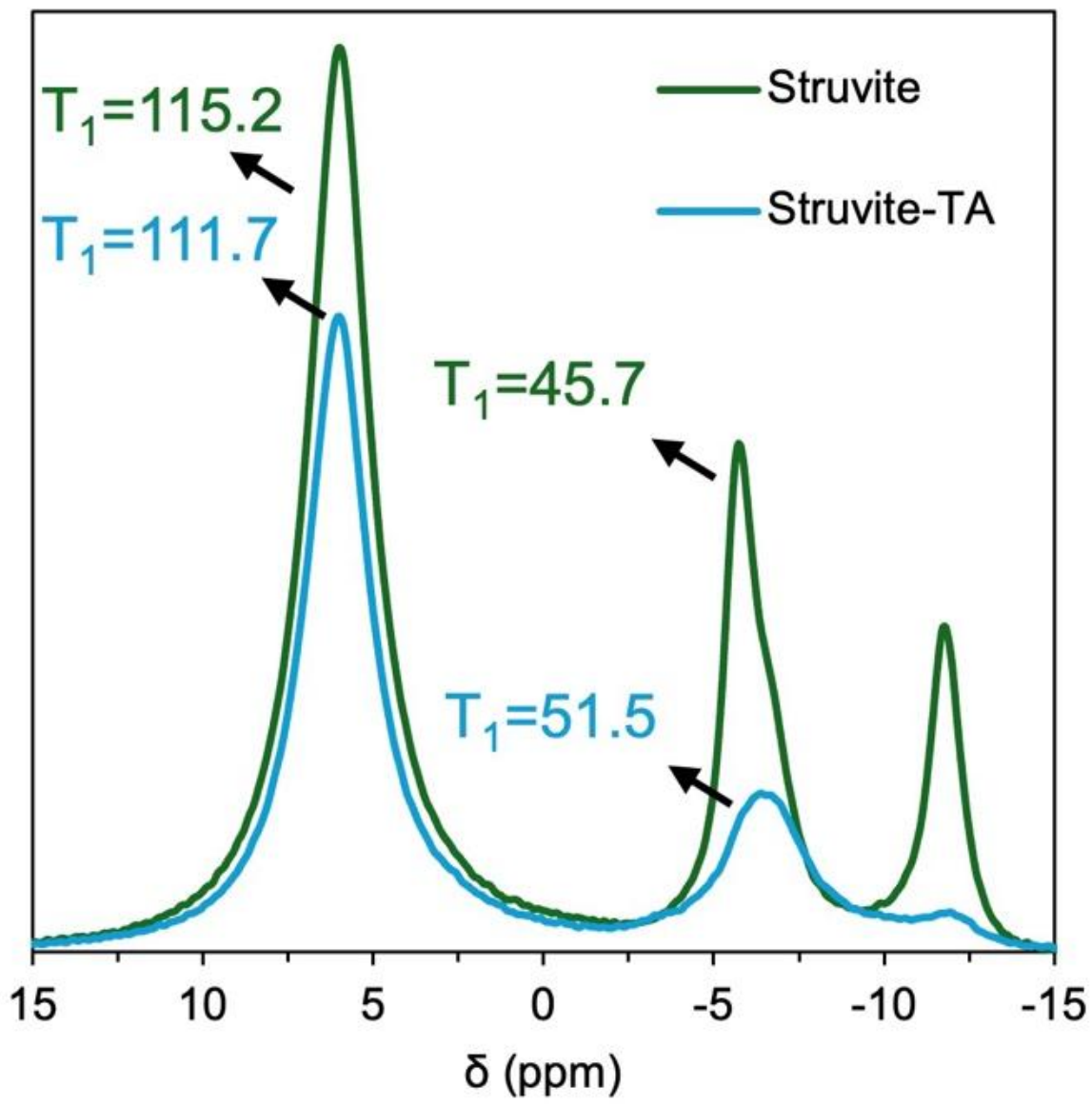

Figure S14. ^{31}P solid-state MAS NMR spectra of struvite and struvite–TA (25%); T_1 is ^{31}P saturation-recovery.

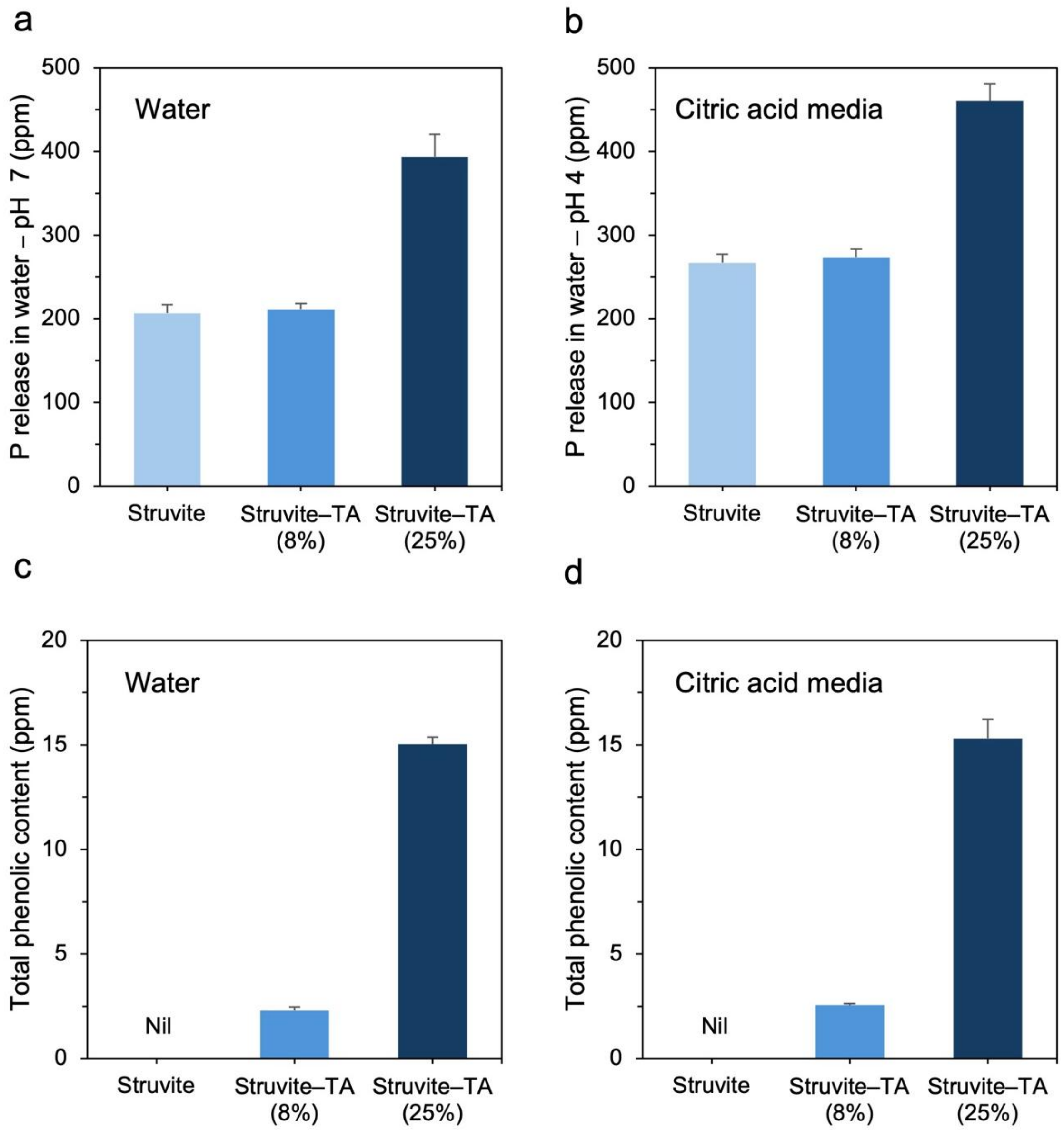

Figure S15. a–d, Release of P (**a,b**) and total phenolic compounds (**c,d**) from struvite or struvite–TA particles in distilled water (pH 6) (**a,c**) and citric acid-acidified water (pH 4) (**b,d**). Data are presented as means ± standard deviations (n = 3).

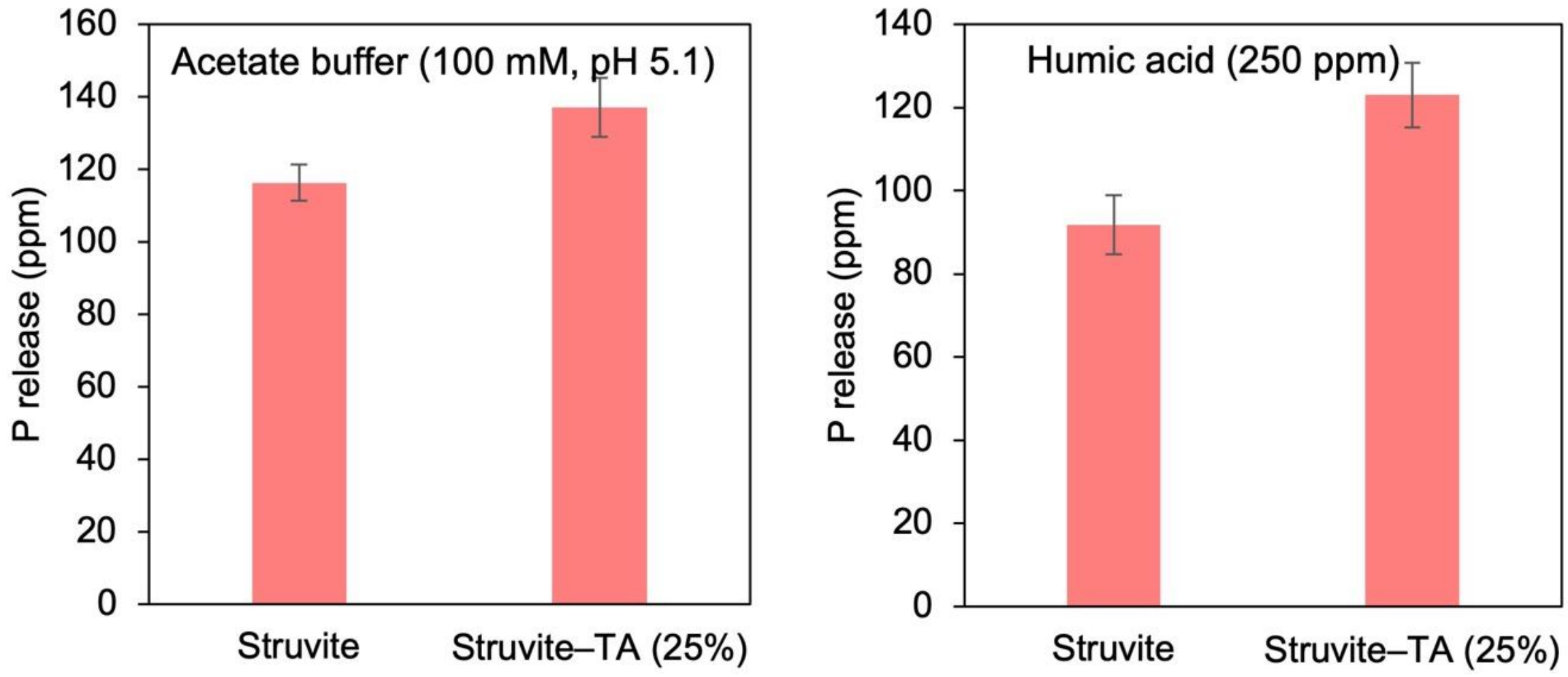

Figure S16. Release of P from struvite and struvite–TA (25%) after 24 h incubation in acetate buffer (100 mM, pH 5.1) and humic acid (250 ppm) solutions. Data are presented as mean ± standard deviation (*n* = 3).

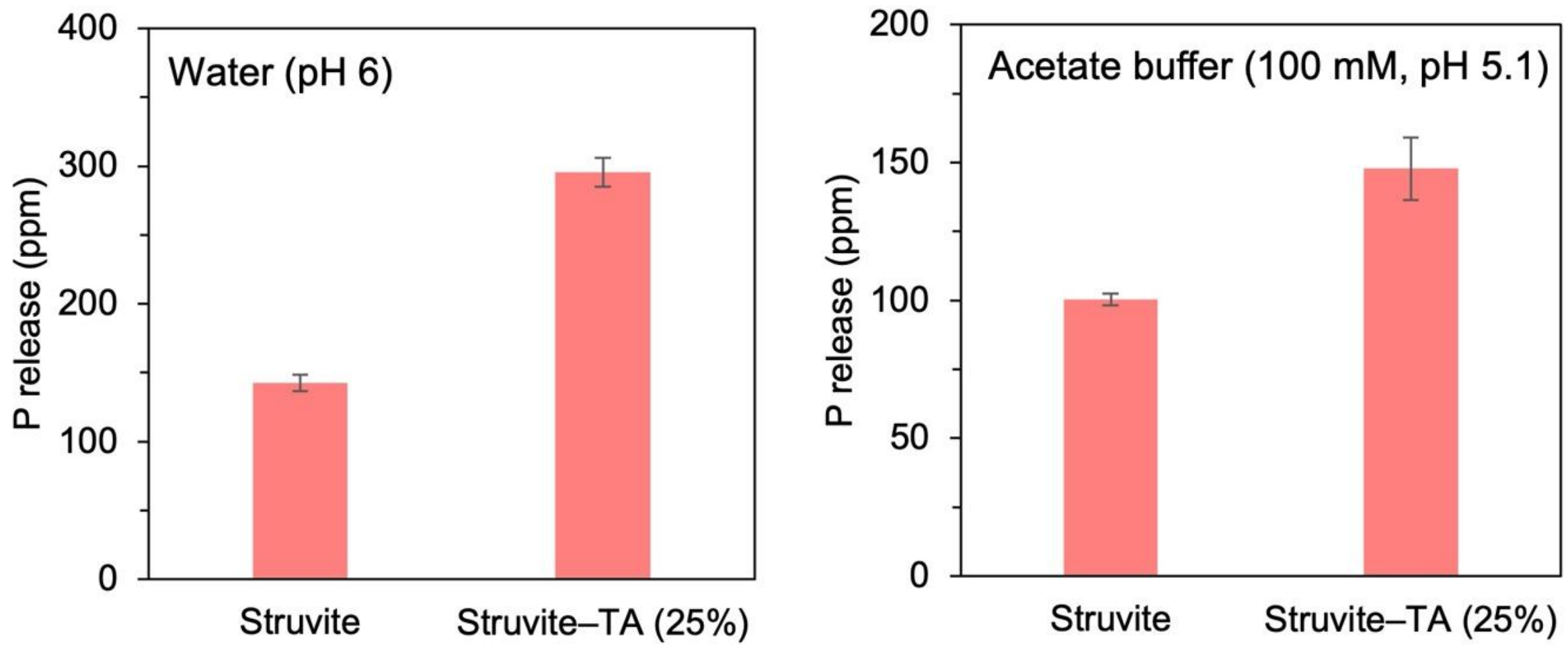

Figure S17. Release of P from struvite and struvite–TA, precipitated at pH 9, in water (pH 6) and acetate buffer media (pH 5.1) after 24 h incubation. Data are presented as mean ± standard deviation ($n = 3$).

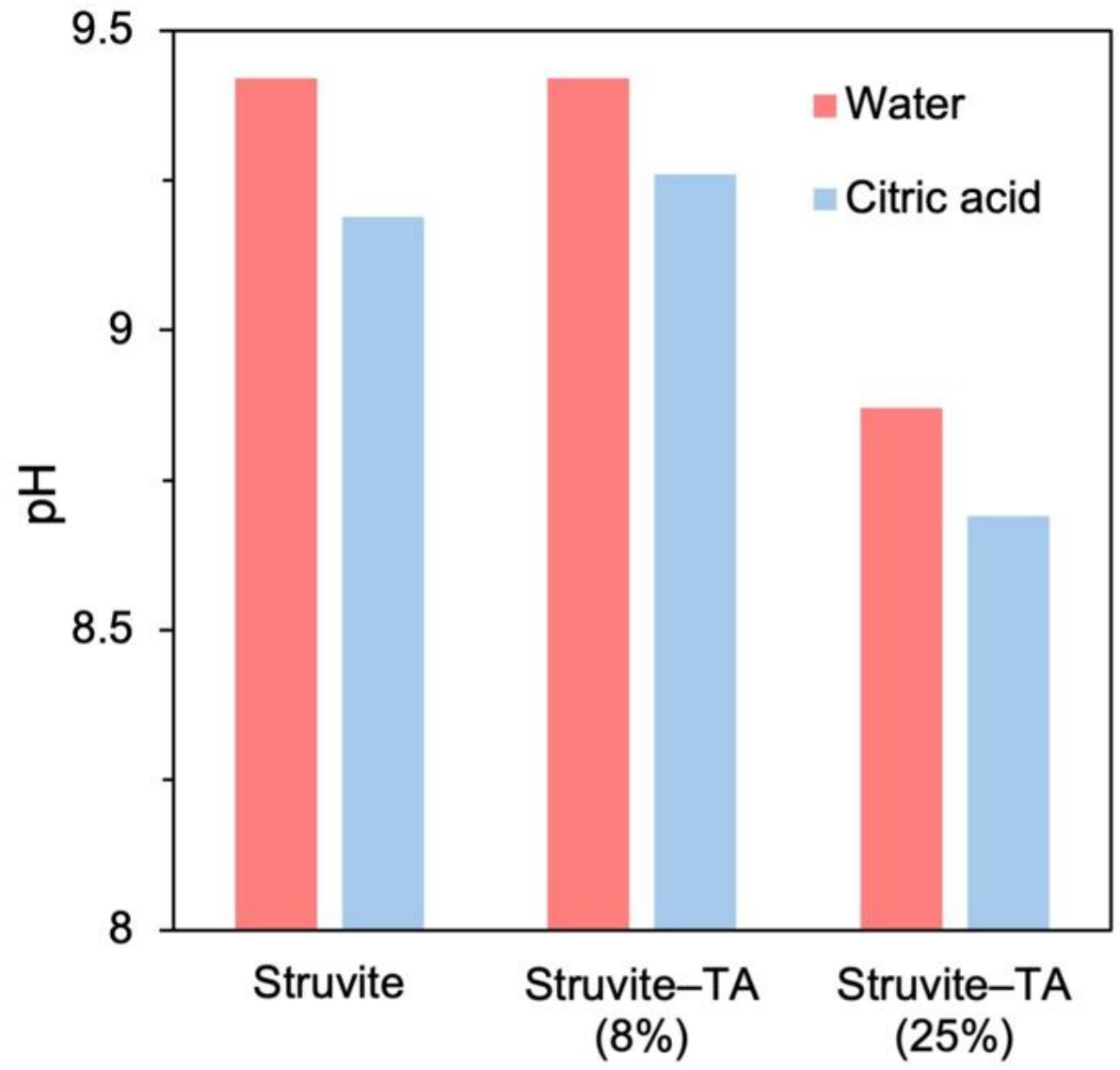

Figure S18. pH values of water and citric acid solutions after incubation of struvite and struvite–TA for 24 h.

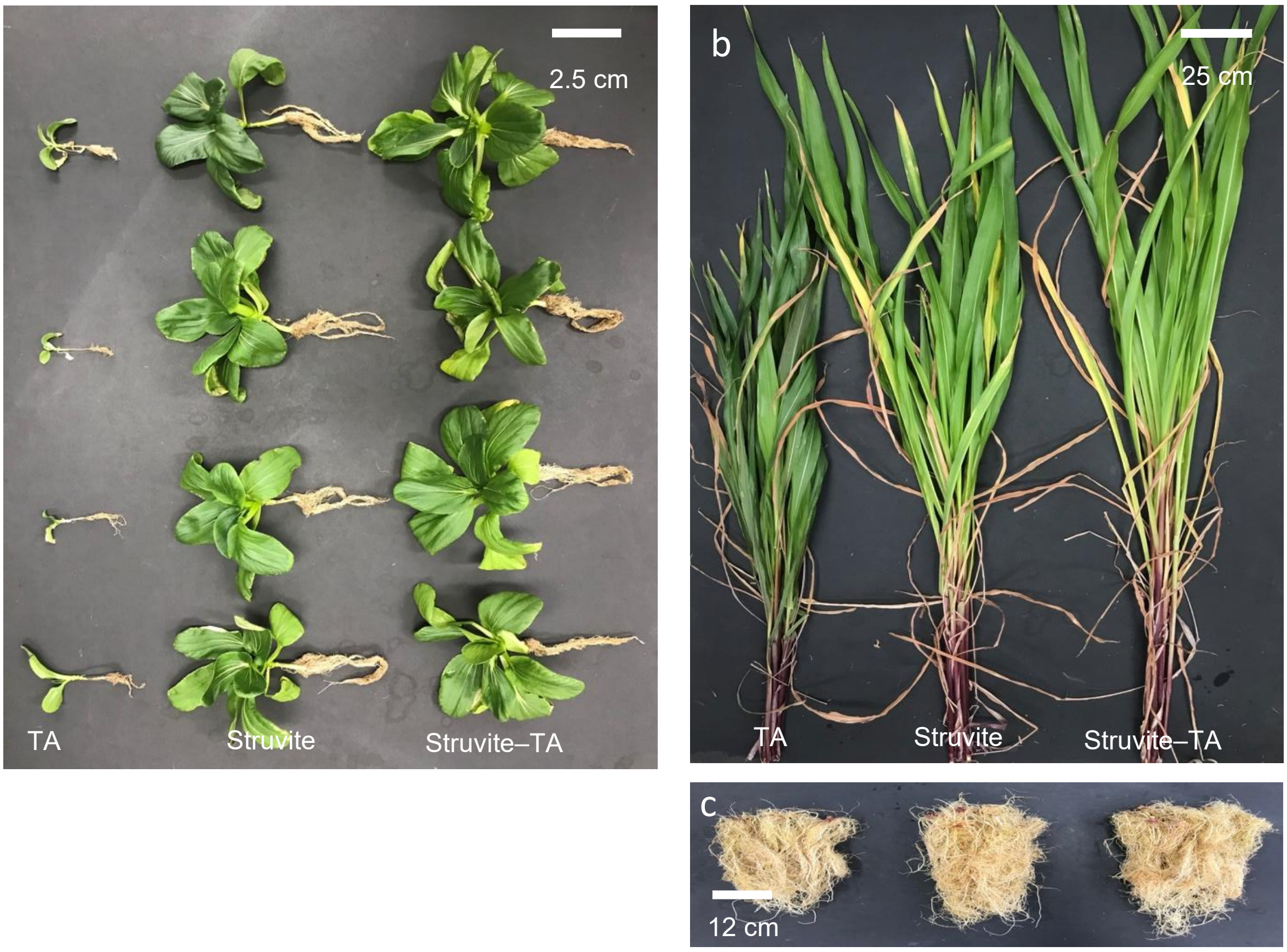

Figure S19. a–c, Photographs of the aboveground parts of pak choi (**a**) and corn (**b**), and corn roots (**c**) grown in soil treated with TA (the same amount of TA as measured in struvite–TA (25%) by TGA was used), struvite particles, or struvite–TA (25%) particles.

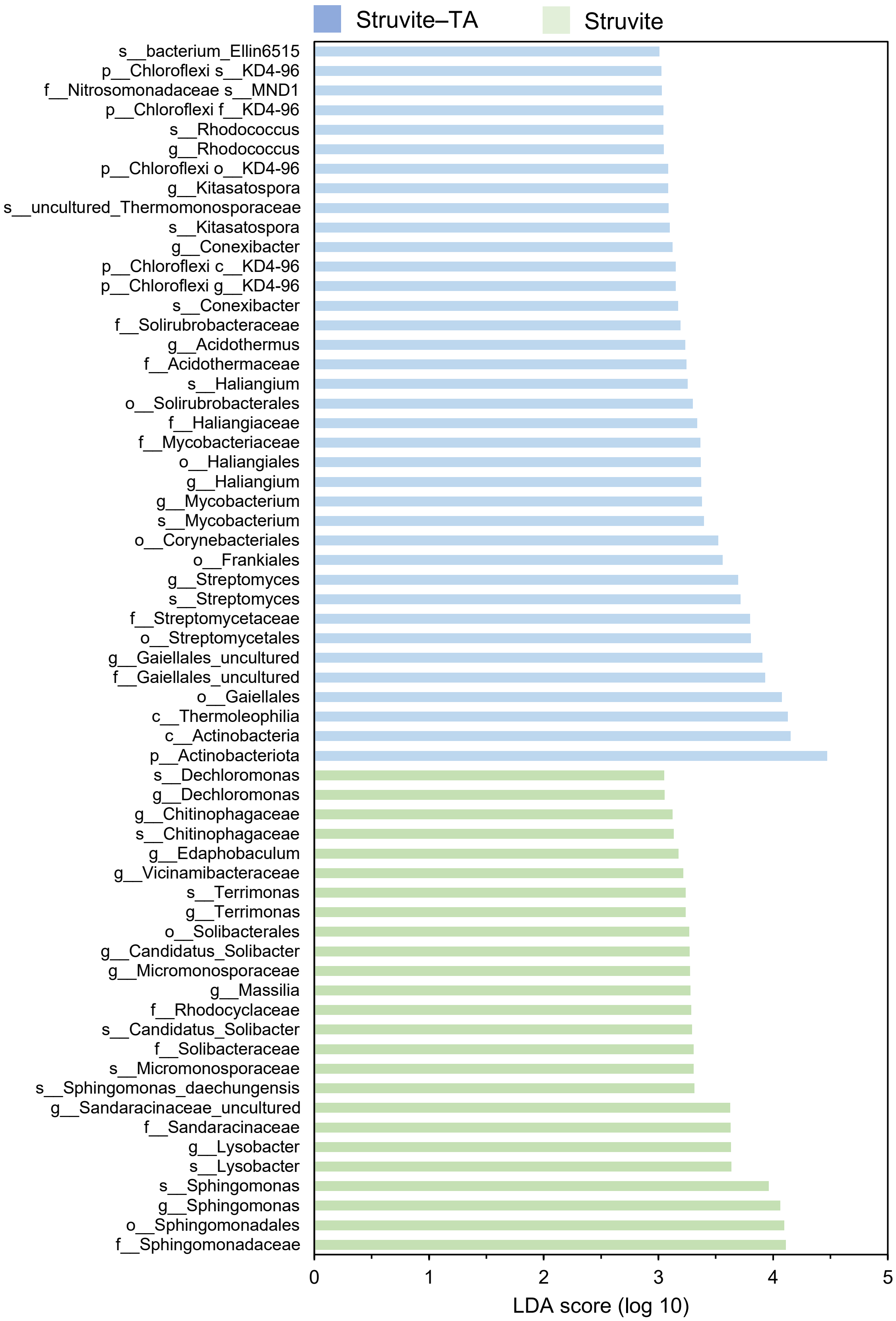

Figure S20. LEfSe analysis (n = 4) identifying soil microbial taxa that are differentially abundant in soils treated with struvite or struvite–TA (25%) particles.

Table S1. Soil properties used in this study[a]

Property	Soil type A (mountain soil collected in Jinju, South Korea)	Soil type B (farmland soil collected in Tatura, Australia)
Organic content	1.88%	4.50%
Ion exchange capacity	12.76 cmol(+)/kg	14.6 cmol(+)/kg
Total-N	0.05%	0.46%
Available P	8.66 mg/kg	130 mg/kg
Electrical conductivity	0.33 ds/m	2.5 ds/m
pH	4.9	5.2

[a]Two types of soils collected from South Korea and Australia were investigated in this study. Lettuce in Figure 4a, 6a, and Figure S8 was grown in soil type B, whereas other crops (Figure 4c,d, 6c,d, and Figure S9) were grown in soil type A. Soil type A was used for leachate experiments (Figure 1).

Table S2. Enriched microbial taxa (see Figure 4e) in soils treated with CaP–TA (8%) and their biological functions[a]

Microbial taxa	Plant growth promoting rhizobacteria	Stress tolerance	Recalcitrant organic matter degradation	Pathogen suppression	References
g__Candidatus_Udaeobacter<br>s__Candidatus_Udaeobacter	■	■		■	- Vasconcellos et al. Current Microbiology 2021, 78, 218–228<br>- Willms et al. Environmental Microbiology Reports 2021, 13, 878–883
g__Lysobacter<br>s__Lysobacter	■			■	- Dai et al. Biology and Fertility of Soils 2023, 59, 317–331
s__Flavisolibacter		■	■		- Liu et al. Biological Control 2016, 101, 103–113<br>- Wilhelm et al. Frontiers in Microbiology 2017, 8, 537
g__env.OPS_17<br>f__env.OPS_17		■	■		- Zhang et al. Water 2023, 15, 2977<br>- Wu et al. Frontiers in Microbiology 2020, 11, 610001
s__KD3-93<br>f__KD3-93<br>g__KD3-93	■				- Wu et al. BMC Microbiology 2022, 22, 232
s__uncultured Sorangiineae	■	■	■	■	- Gerth et al. The Journal of Antibiotics 1994, 47, 23–31<br>- Gerth et al. The Journal of Antibiotics 1996, 49, 560–563<br>- Mohr et al. International Journal of Systematic and Evolutionary Microbiology 2018, 68, 3576–3586
s__Nitrospira japonica	■	■			- Ushiki et al. Journal of Bioscience and Bioengineering 2017, 123, 581–589
g__Adhaeribacter	■		■		- Gilbert and Rickard, Bergey's Manual of Systematics of Archaea and Bacteria, Adhaeribacter, 2015<br>- Chhetri et al. International Journal of Systematic and Evolutionary Microbiology 2020, 70, 5382–5388<br>- Yu et al. Rhizosphere 2025, 33, 101047<br>- Degrune et al. Frontiers in Microbiology 2017, 8, 1127

[a]Reported cases in the literature are indicted by the blue-colored cells.

Table S3. Enriched microbial taxa (see Figure 6e) in soils treated with struvite–TA (25%) and their biological functions[a]

Microbial taxa	Plant growth promoting rhizobacteria	Stress tolerance	Recalcitrant organic matter degradation	Pathogen suppression	References
o__Sphingomonadales<br>f__Sphingomonadaceae<br>g__Sphingomonas<br>s__Sphingomonas<br>s__Sphingomonas_daecheungensis					- Luo et al. Frontiers in Microbiology 2019, 10, 1221<br>- Lombardino et al. Microbiology Spectrum 2022, 10, e01994-21
g__Lysobacter<br>s__Lysobacter					- Dai et al. Biology and Fertility of Soils 2023, 59, 317–331
f__Sandaracinaceae<br>g__Sandaracinaceae_uncultured					- Saraf and Sharma, Frontiers in Microbiology 2025, 16, 1550287
o__Solibacterales<br>f__Solibacteraceae<br>g__Candidatus__Solibacter<br>s__Candidatus__Solibacter					- Mastný et al. Scientific Reports 2021, 11, 18677<br>- Kalam et al. Frontiers in Microbiology 2020, 11, 580024
f__Rhodocyclaceae					- Fernández-Llamosas et al. Microorganisms 2020, 8, 1453<br>- Becker et al. Microbial Physiology 2024, 34, 1–77
f__Micromonosporaceae<br>g__Micromonosporaceae					- Martínez-Hidalgo et al. Scientific Reports 2014, 4, 6389
g__Terrimonas<br>s__Terrimonas					- Terrapon et al. Nucleic Acids Research 2018, 46, 677–683
f__Vicinamibacteraceae					- Yu et al. Agronomy 2025, 15, 1548
g__Edaphobaculum					- Mayer et al. Applied Soil Ecology 2026, 217, 106656
g__Massilia					- Chhetri et al. Chemosphere 2024, 368, 143776<br>- Li et al. International Journal of Systematic and Evolutionary Microbiology 2021, 71, 005009.<br>- Amirhosseini et al. Microbial Biotechnology 2025, 18, e70156
g__Chitinophagaceae<br>s__Chitinophagaceae					- Kim et al. Scientific Reports 2024, 14, 1038<br>- Rosenberg, The Prokaryotes, The Family Chitinophagaceae, 2014, 493–495
g__Dechloromonas<br>s__Dechloromonas					- Lee and Cho, Journal of Microbiology and Biotechnology 2024, 34, 2252–2265<br>- Zhou et al. Nature Communications 2025, 16, 5102.

[a]Reported cases in the literature are indicted by the blue-colored cells.
